# A novel biologically hierarchical hydrogel with osteoblast precursor‐targeting extracellular vesicles ameliorates bone loss in vivo via the sequential action of antagomiR‐200b‐3p and antagomiR‐130b‐3p

**DOI:** 10.1111/cpr.13426

**Published:** 2023-02-14

**Authors:** Hanhao Dai, Yunlong Yu, Junyong Han, Jun Luo, Chao Song, Zhibo Deng, Yijing Wu, Dianshan Ke, Jie Xu

**Affiliations:** ^1^ Shengli Clinical Medical College of Fujian Medical University Fuzhou China; ^2^ Department of Orthopedics Fujian Provincial Hospital, Fujian Medical University Fuzhou China; ^3^ Institute for Immunology, Fujian Academy of Medical Sciences Fuzhou China

## Abstract

Osteoporotic fracture is a major health problem plaguing the ageing society, and improving its treatment is an urgent challenge. How to ameliorate bone loss determines the recovery of such fractures. Extracellular vesicle (EV)‐loaded hydrogel has the capacity to treat osteoporotic fractures due to its pro‐osteogenic property. And balancing proliferation and maturation of osteoblast precursors (OBPs) is of great significance to avoid OBP depletion, which is lacking in current treatment. Based on osteoblastogenic miRNAs, this study aimed to explore the efficacies of the combination of hierarchical hydrogel and EVs altering functional miRNAs level in bone loss. Through bioinformatics analyses, we screened out proliferative gene‐targeting miR‐200b‐3p and osteogenic gene‐targeting miR‐130b‐3p. And antagomiR‐200b‐3p (ant‐200b) enhanced OBP proliferation, and antagomiR‐130b‐3p (ant‐130b) promoted OBP differentiation. After confirming the directional effect of Fibronectin (Fn1) on OBPs, we prepared OBP‐targeting EVs. Furthermore, encapsulation of two antagomiRNAs in EVs enhanced the respective effect of ant‐200b and ant‐130b. Notably, hierarchically injectable hydrogel exerted an effective function in promoting the sequential delivery of EVs‐200b and EVs‐130b. Importantly, hierarchical hydrogel containing dual EVs effectively ameliorated bone loss. Overall, hierarchical hydrogel based on two antagomiRNAs effectively improves bone loss in vivo due to its role in promoting OBP proliferation and maturation sequentially.

## Graphical abstract

The working model diagram regarding preparation and therapeutic effect of hierarchically injectable hydrogel in this study. In short, antagomiRNAs (ant‐130b and ant‐200b) based on the two screened miRNAs are transfected into Fn1‐overexpressing EVs (Fn1‐EVs), which form EVs‐130b and EVs‐200b. Subsequently, a hierarchically injectable hydrogel with the dual EVs is constructed, which contains SA in the inner layer and PF‐127 in the outer layer. Then, OVX osteoporosis mice receive the paraperiosteal injection of the above hydrogel, and dual EVs are directional released into OBPs in vivo in turn. OBP proliferation is firstly promoted by the enhanced Sox2 expression with EVs‐200b, and then osteoblastic osteogenesis is promoted by the enhanced Runx2 expression with EVs‐130b. The above treatment regimen effectively maintains more OBP reserves and improves bone loss in OVX mice.

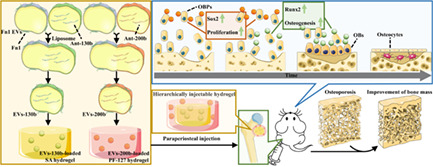

## 1 INTRODUCTION

As a systemic skeletal disorder, osteoporosis is characterized by progressive bone destruction and low bone mass.^1^ Osteoporotic fracture is a high‐risk factor causing disability and death, which has resulted in serious social problems.^1^ Osteoporosis‐related fractures, including hip and spinal fractures, have a significant impact on patients' daily activities.^2^ Moreover, patients with hip fractures have a two‐fold increased risk of mortality.^3^ And vertebral fractures also pose a serious threat to the survival of patients.^4^ Notably, the mortality rate of patients with secondary fractures has increased by approximately 80%–90%.^3^ According to statistics, the fracture caused by osteoporosis is as high as 30% in the women and 11% in the men ≥50 years, which has buried a major hidden danger for the aging society.^3^ Bone health depends on the dynamic balance between bone formation dominated by osteoblasts and bone absorption dominated by osteoclasts.^1^ The destruction of the above balance can lead to the degradation of bone fibres and increased bone fragility, eventually leading to osteoporosis and subsequent fractures.^1^ Therefore, the prevention and treatment of bone loss is the key to improve osteoporotic fracture. Previous studies have demonstrated that the increase of bone mineral density (BMD) in the hip, femoral neck and vertebra can effectively prevent hip and vertebral fractures, and reduce the risk of disability and death in patients with osteoporosis.^5^, ^6^, ^7^, ^8^ Accordingly, in the treatment of osteoporotic fractures, more attention should be paid to low bone mass. Biomaterials‐related therapeutic strategies that can increase BMD locally are urgently required.

Numerous studies have shown that extracellular vesicles (EVs)‐loaded hydrogels can locally improve osteogenesis, thereby enhancing bone reconstruction. Wu et al. encapsulated bone mesenchymal stem cells (BMSC)‐derived EVs into the chitosan/β‐glycerophosphate hydrogel to repair rat calvarial defects.^9^ In addition, several studies reported that MSC‐derived EVs are loaded into different injectable hydrogels, resulting in improving angiogenesis and osteogenesis, which was used to promote bone regeneration in rat bone defect models.^10^, ^11^, ^12^, ^13^ To our knowledge, few studies reported the application of EV‐loaded injectable hydrogels in the improvement of hip or vertebral BMD. Therefore, based on EV‐loaded hydrogels, it is of great scientific significance to seek a local improvement scheme for BMD to prevent hip and vertebral fractures.

Modifying EVs by altering the cargos in EVs is an effective approach to enhance the therapeutic effects of EVs. Hydrogel‐based nano‐biomaterials can be used to load and deliver osteoporosis drug parathyroid hormone (PTH), which was reported in several studies regarding bone regeneration.^14^, ^15^, ^16^ PTH has significant efficacy in treating osteoporosis due to its pro‐osteogenic property. Thus, PTH has been widely used in clinical treatment. However, some disadvantages exist in PTH treatment. Long‐term application of PTH may cause several side effects, including nausea, leg cramps, and osteosarcoma.^17^, ^18^, ^19^ Also, withdrawal of PTH can lead to abnormal enhancement of adipogenic differentiation of BMSCs.^20^ Most importantly, PTH can only promote the osteogenic differentiation while having no effects on the proliferation of osteoblast precursors (OBPs),^21^, ^22^, ^23^ thus resulting in the exhaustion of OBPs, which becomes a serious disadvantage in PTH treatment. Following the maturation from OBPs to osteoblasts (OBs), how to retain enough OBPs has become a significant challenge. Accordingly, the therapeutic strategy on balancing the proliferation and maturation of OBPs has become a more perfect scheme for the treatment of osteoporosis.

Some microRNAs (miRNAs) are known to have the capacity to regulate the osteogenesis and proliferation of OBPs. Several miRNAs can suppress the differentiation of OBPs to mature OBs, all of which are attributed to the combination of the 3′‐untranslated region (3′‐UTR) of runt‐related transcription factor 2 (RUNX2).^24^, ^25^, ^26^ Furthermore, the synthesis of miR‐188 inhibitor into EVs can effectively improve the repressed osteogenesis caused by corresponding miRNA.^27^ In addition, some other miRNAs are responsible for the reduction in the proliferation of OBPs by targeting corresponding proliferation‐related genes.^28^, ^29^, ^30^, ^31^, ^32^ Accordingly, screening novel miRNAs that could regulate the proliferation and osteogenic differentiation of OBPs by using bioinformatics methods is essential for balancing the proliferation and maturation of OBPs during the treatment of osteoporosis.

Sex‐determining region Y‐box 2 (Sox2) and Runx2 are key genes regulating the proliferation and osteogenic differentiation of OBPs,^33^, ^34^ respectively. In this study, we identified that Sox2‐targeting miRNA, miR‐200b‐3p, and Runx2‐targeting miRNA, miR‐130b‐3p, were differentially upregulated in senescent OBPs. And miR‐200b‐3p inhibition by antagomiR‐200b‐3p (ant‐200b) could improve the proliferation of OBPs, while miR‐130b‐3p inhibition by antagomiR‐130b‐3p (ant‐130b) was conducive to the differentiation of OBPs to mature OBs. Relying on the finding that fibronectin1 (Fn1) on the surface of BMSC‐derived EVs could bind to the membrane proteins of OBPs, we prepared OBP‐targeting EVs by overexpressing Fn1 in MSCs. Subsequently, ant‐200b and ant‐130b were respectively transduced in OBP‐targeting EVs to construct ant‐200b‐loaded and ant‐130b‐loaded EVs (EVs‐200b and EVs‐130b). The roles of EVs‐200b and EVs‐130b on OBPs were evaluated in vitro. In in vivo assays, we prepared a hierarchically injectable hydrogel with sodium alginate (SA) in the inner layer and Pluronic F‐127 (PF‐127) in the outer layer to sequentially release EVs‐200b and EVs‐130b, which successively improve the proliferation and osteogenic differentiation of OBPs. Overall, our study presented a novel biomaterial for optimizing the improvement of BMD in the designed site.

## 2 MATERIALS AND METHODS

A detailed description of all materials and methods can be found in Supporting Information S1: materials and methods.

## 3 RESULTS

### 3.1 Prediction of differentially expressed miRNAs targeting proliferative and osteogenic genes

**FIGURE 1:**
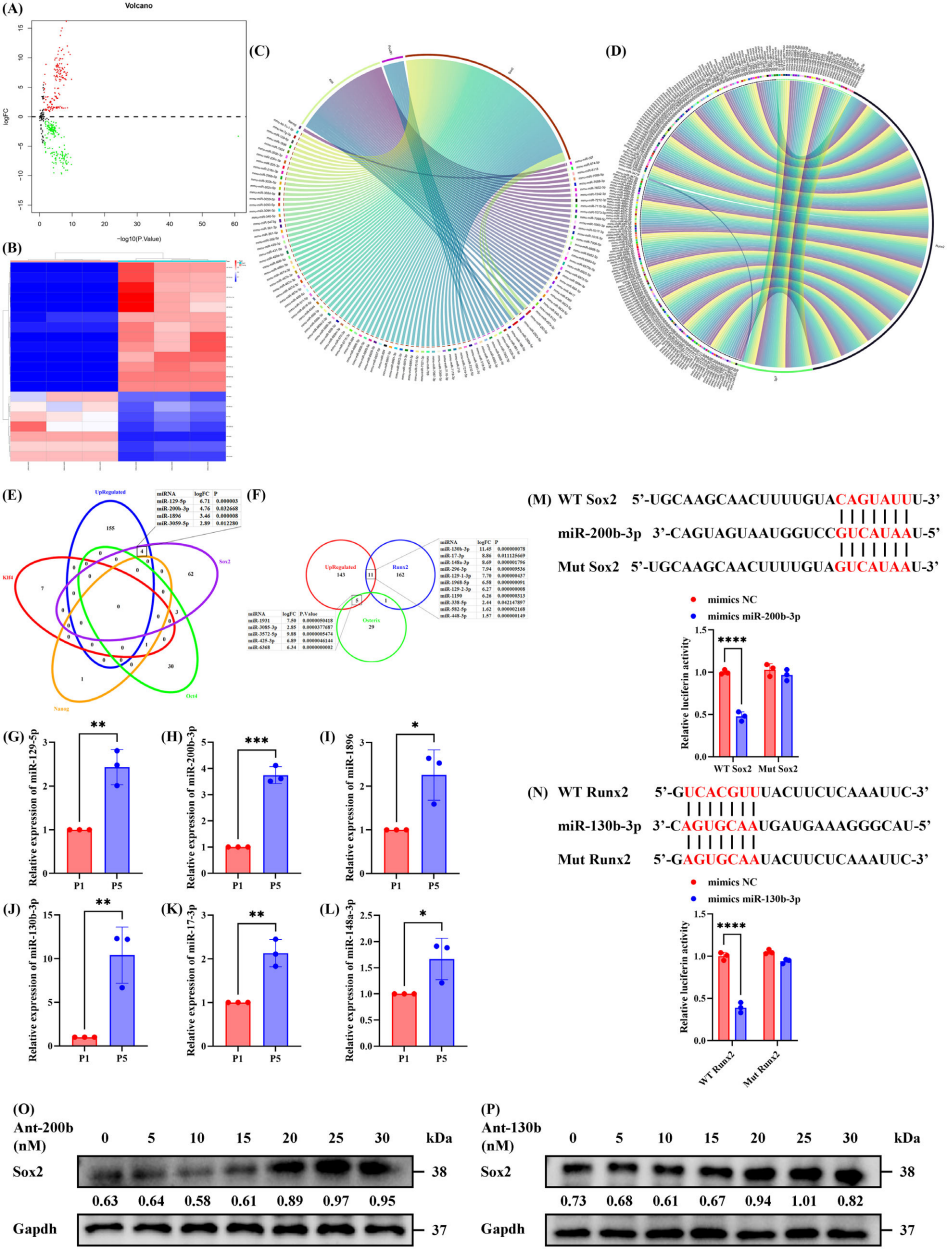
Prediction of differential miRNAs targeting proliferative and osteogenic genes. (A) Volcano plot of differentially expressed miRNAs in young and old BMSCs. (B) Hierarchical clustering heatmap (red: high expression, white: medium expression and blue: low expression) of differentially expressed miRNAs in young and old BMSCs. (C) Chord diagram of the predicted miRNAs and the indicated proliferative genes. (D) Chord diagram of the predicted miRNAs and the indicated osteogenic differentiated genes. (E) Venn diagram of the upregulated miRNAs and the predicted miRNAs with the ability to interact with the indicated proliferative genes. (F) Venn diagram of the upregulated miRNAs and the predicted miRNAs with the ability to interact with the indicated osteogenic differentiated genes. (G–L) The levels of miR‐129‐5p (G), miR‐200b‐3p (H), miR‐1896 (I), miR‐130b‐3p (J), miR‐17‐3p (K) and miR‐148a‐3p (L) in the P1 and P5 OBPs were determined by qRT‐PCR. (M) The luciferase activity of the WT Sox2 and Mut Sox2 in 293 T cells treated with mimics NC or mimics miR‐200b‐3p. (N) The luciferase activity of the WT Runx2 and Mut Runx2 in 293 T cells treated with mimics NC or mimics miR‐130b‐3p. (G–L) *n* = 3. Values are shown as mean ± SD. *p < 0.05, **p < 0.01, ***p < 0.001, Student's *t* test. (M, N) *n* = 3. Values are shown as mean ± SD. ****p < 0.0001, two‐way ANOVA. ANOVA, analysis of variance; BMSC, bone mesenchymal stem cell; NC, negative control; OBP, osteoblast precursor; P1, the first generation of OBPs; P5, the fifth generation of OBPs; WT, wild type; Mut, mutant type.

First, we needed to screen out specific miRNAs having the capacity to regulate the proliferation and osteogenic differentiation of OBPs. Due to the lack of OBP‐related databases, we conducted relevant bioinformatics analyses on homogenetic OBP‐derived cells and performed in vitro functional verification. The differentially expressed miRNAs in young and aged BMSCs were identified through bioinformatics analysis. It was found that compared with young BMSCs, 159 miRNAs were upregulated, whereas 184 miRNAs were downregulated in aged BMSCs in GSE57127 with a |log_2_ FC| cutoff criteria >0.5 and *p* value < 0.05 (Figure 1A,B). Next, the proliferative gene‐targeting miRNAs (Figure 1C) and osteogenic gene‐targeting miRNAs (Figure 1D) were predicted using miRWalk 3.0, TargetScan and miRDB database, and the gene‐miRNA pairs were visualized using chord diagrams. Then, the abnormally upregulated miRNAs in aged BMSCs were overlapped with the proliferative gene‐targeting miRNAs (Figure 1E) and the osteogenic gene‐targeting miRNAs (Figure 1F). Subsequently, qRT‐PCR was performed to evaluate the levels of top 3 upregulated proliferative gene‐targeting miRNAs and osteogenic gene‐targeting miRNAs in P1 and P5 OBPs. Detection of proliferative gene‐targeting miRNAs showed that the levels of miR‐129‐5p (about 2.4 times; Figure 1G), miR‐200b‐3p (about 3.7 times; Figure 1H) and miR‐1896 (about 2.3 times; Figure 1I) in P5 OBPs were greater than those in P1 OBPs. And the upregulation of osteogenic gene‐targeting miRNAs was also confirmed: miR‐130b‐3p (about 10.4 times; Figure 1J), miR‐17‐3p (about 2.1 times; Figure 1K) and miR‐148a‐3p (about 1.7 times; Figure 1L). As the two most upregulated miRNAs, miR‐200b‐3p and miR‐130b‐3p were selected for further investigation. As shown in the dual‐luciferase assays, miR‐200b‐3p mimics and miR‐130b‐3p mimics significantly inhibited the luciferase activity of the WT Sox2 and WT Runx2, respectively. By treating OBPs with gradient concentration of ant‐130b and ant‐200b, we chose 20 nM as the optimal therapeutic concentration because the highest three concentrations have similar upregulation effects, and the lowest of them is more appropriate (Figure 1O,P).

To verify the reliability of this experimental system, it was necessary to evaluate the proliferative and osteogenic abilities of P5 OBPs. β‐gal senescence‐related staining showed that there existed more β‐gal positive cells in P5 OBPs than in P1 OBPs (Figure S1A,B), which confirmed the senescence of P5 OBPs. And the protein level of p16, a marker of irreversible senescence, was markedly increased in P5 OBPs, while the protein expression of Sox2 was significantly decreased in P5 OBPs (Figure S1C). We found that the proliferative potential was decreased in P5 OBPs presented as less cell viability in CCK8 assay (Figure S1D), a lower percentage of cells in the S phase in FCM assay (Figure S1E,F), and fewer EdU‐positive cells (Figure S1G,H). Also, the fluorescence intensity of Ki67, a recognized proliferative marker, significantly declined in P5 OBPs compared with P1 OBPs (Figure S1I,J). Similarly, P5 OBPs had lower protein levels of Runx2, Col1a1, Ocn and Opn than P1 OBPs (Figure S2A). And ARS staining and ALP staining showed that P5 OBPs displayed lower calcification levels and ALP activity, respectively (Figure S2B–E). In addition, IF staining also showed that the fluorescence intensity of Runx2, Col1a1, Ocn and Opn were obviously downregulated in P5 OBPs (Figure S2F–M).

### 3.2 Ant‐200b and ant‐130b, respectively, improved the proliferation and osteogenesis of senescent OBPs by targeting Sox2 and Runx2

**FIGURE 2:**
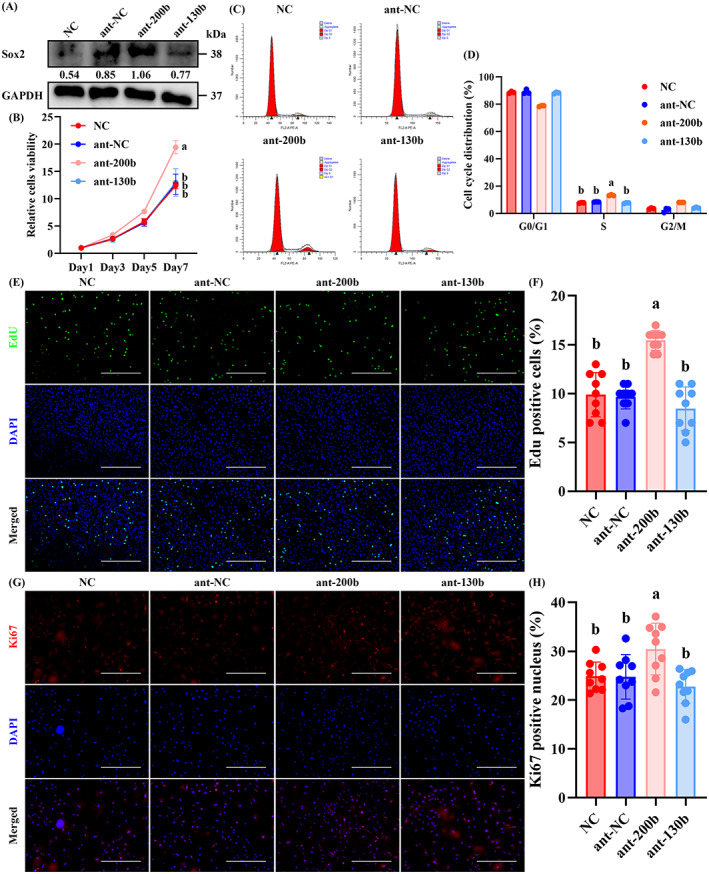
Ant‐200b improved the proliferation of senescent OBPs by targeting Sox2. (A) Western blotting of Sox2 level in the NC, ant‐NC, ant‐200b and ant‐130b groups. GAPDH was used as a loading control. The values below the band represent the ratio of the grey value of Sox2 and GAPDH by ImageJ. (B) CCK8 assays for cell proliferation of each group. (C) FCM for the cell cycle of each group. (D) Quantitative analyses of OBPs in the G0/G1 phase, S phase and G2/M phase among each group. (E) Representative EdU staining of each group. (F) Quantitative analyses of EdU‐positive cells. (G) Representative IF staining for Ki67 of each group. (H) Quantitative analyses of Ki67‐positive cells. (B) *n* = 3. Values are shown as mean ± SD. ^Letter^ *p* <0.05, two‐way ANOVA. (D) *n* = 3. Values are shown as mean ± SD. ^Letter^ *p* <0.05, one‐way ANOVA. (F, H) *n* = 3, three fields per sample were selected. Values are shown as mean ± SD. ^Letter^ *p* <0.05, one‐way ANOVA. (E) Scale bar = 200 μm. (G) Scale bar = 100 μm. ANOVA, analysis of variance; ant‐NC, antagonist of empty carrier; NC, negative control; OBP, osteoblast precursor.

Subsequently, we aimed to investigate the effect of miR‐200b‐3p or miR‐130b‐3p on the proliferation and osteogenic differentiation of senescent OBPs by using ant‐200b and ant‐130b, respectively. Sox 2 protein expression was significantly enhanced in ant‐200b‐treated P5 OBPs, but was not affected in ant‐130b‐treated P5 OBPs (Figure 2A). It was found that the proliferative potential was increased in ant‐200b‐treated P5 OBPs presented as stronger cell viability in CCK8 assay (Figure 2B), a higher percentage of cells in S phase in FCM assay (Figure 2C,D), and more EdU‐positive cells (Figure 2E,F). IF training also showed that Ki67 fluorescence intensity was the highest in the ant‐200b group (Figure 2G,H). Nevertheless, the above proliferative parameters did not change in ant‐130b‐treated P5 OBPs (Figure 2A–H).

**FIGURE 3:**
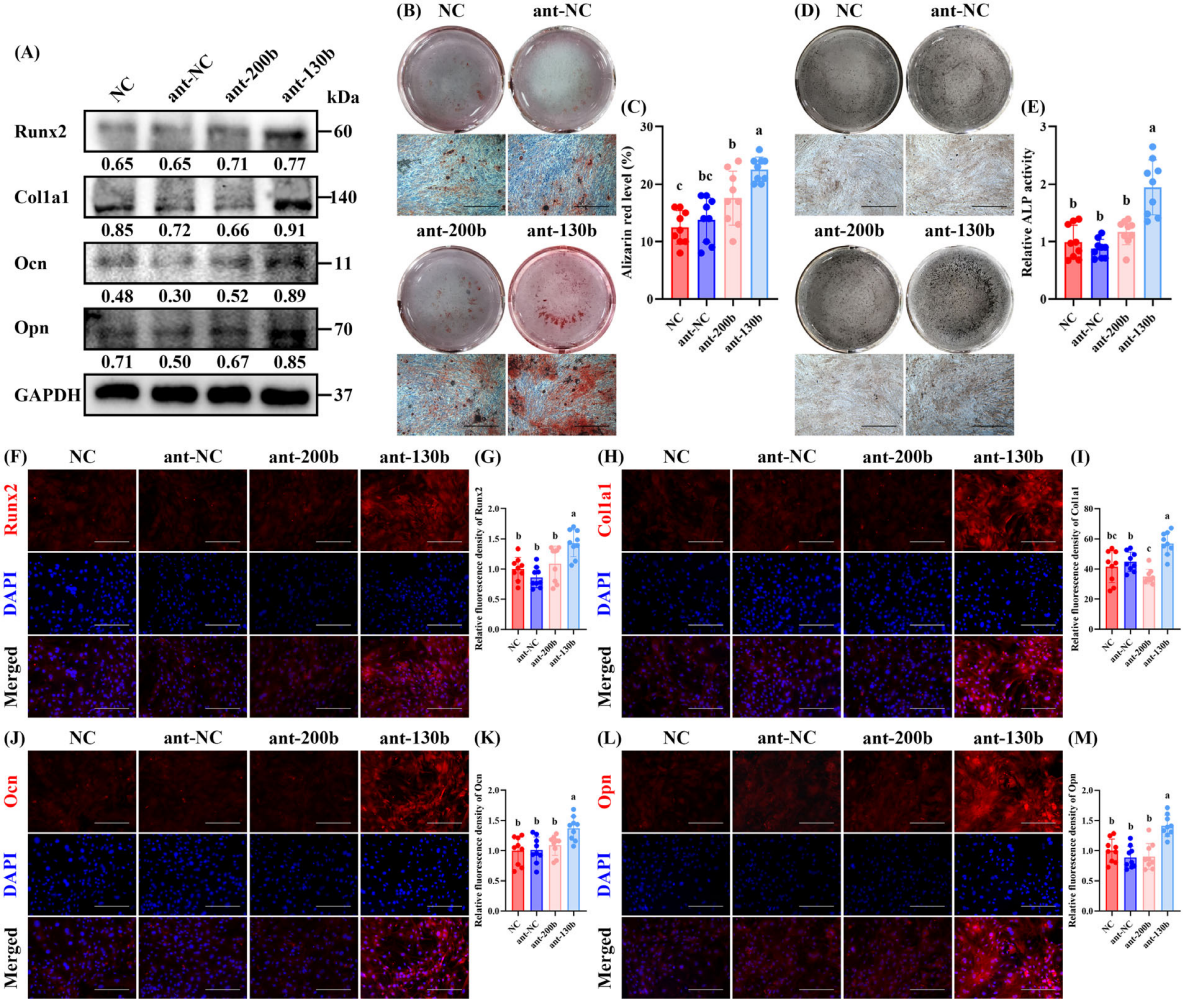
Ant‐130b improved the osteogenesis of senescent OBPs by targeting Runx2. (A) Western blotting of Runx2, Col1a1, Ocn and Opn levels in the NC, ant‐NC, ant‐200b and ant‐130b groups. GAPDH was used as a loading control. The values below the band represent the ratio of the grey value of target proteins and GAPDH by ImageJ. (B) Representative ARS staining of each group. (C) Quantitative analyses of ARS level. (D) Representative ALP staining of each group. (E) Quantitative analyses of ALP activity. (F–M) Representative IF staining for Runx2 (F), Col1a1 (H), Ocn (J) and Opn (L) of each group, and quantitative analyses of Runx2 (G), Col1a1 (I), Ocn (K) and Opn (M)‐positive cells. (C, E, G, I, K and M) *n* = 3, three fields per sample were selected. Values are shown as mean ± SD. ^Letter^ *p* <0.05, one‐way ANOVA. (B, D) Scale bar = 200 μm. (F, H, J and L) Scale bar = 50 μm. ant‐NC, antagonist of empty carrier; NC, negative control; OBP, osteoblast precursor.

In addition, the osteogenic differentiation of P5 OBPs was significantly improved by ant‐130b treatment presented as the enhanced expression of osteogenic markers (Runx2, Col1a1, Ocn and Opn) (Figure 3A), more obvious calcification (Figure 3B,C), and stronger ALP activity (Figure 3D,E). IF staining also showed that the fluorescence intensity of Runx2 (Figure 3F,G), Col1a1 (Figure 3H,I), Ocn (Figure 3J,K), and Opn (Figure 3L,M) in P5 OBPs were upregulated by ant‐130b treatment. Ant‐200b treatment also showed a higher calcification level, which was significantly lower than that of the ant‐130b group. In addition, ant‐200b treatment slightly promoted ALP activity as well as the fluorescence intensity of Runx2 and Ocn in P5 OBPs, all of which had no statistical significance. Other osteogenic parameters were not affected by ant‐200b treatment (Figure 3A–M).

### 3.3 Encapsulation of antagomiRNAs in OBP‐targeting EVs enhanced the respective effect of ant‐200b and ant‐130b

**FIGURE 4:**
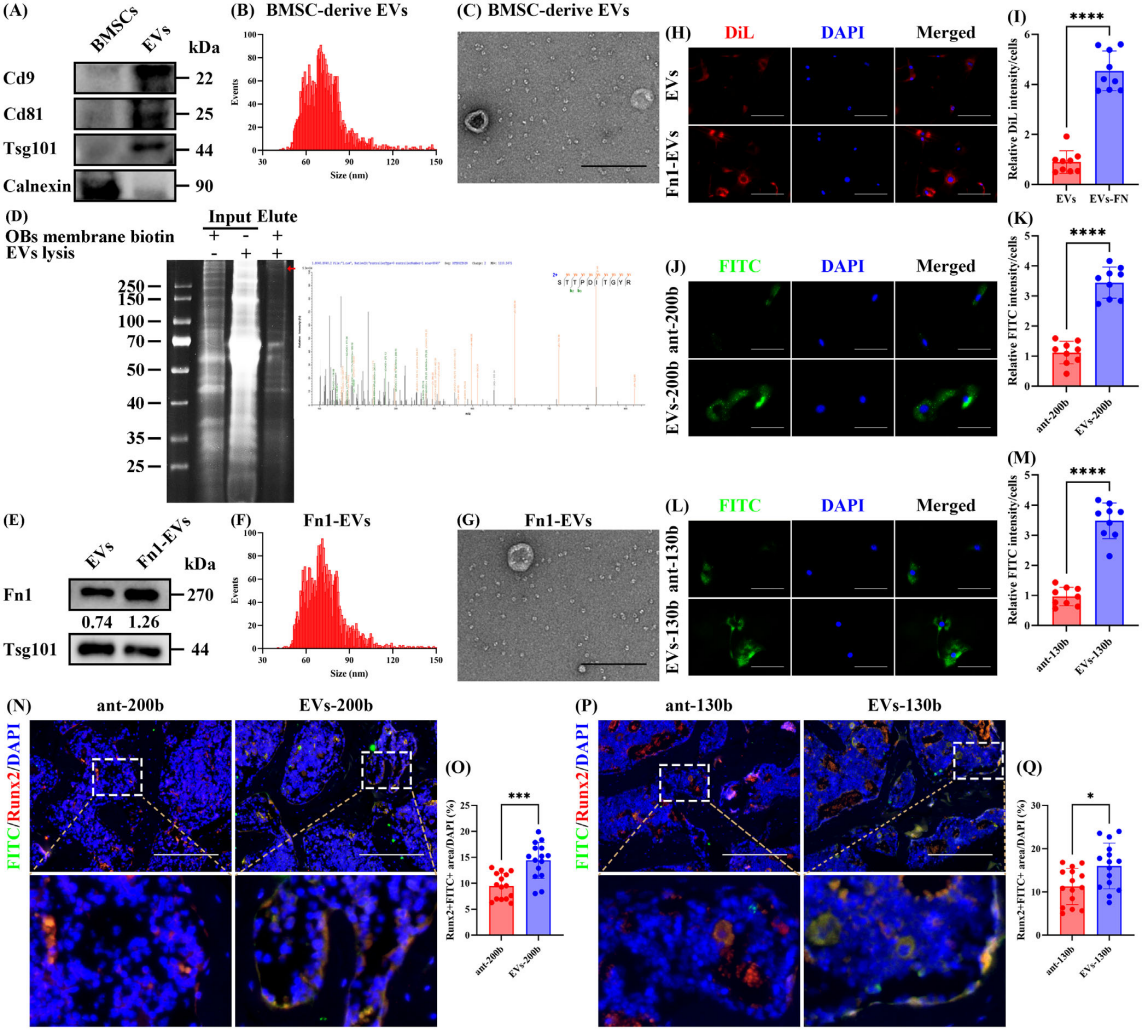
Encapsulation of antagomiRNAs in OBP‐targeting EVs improved their transfection efficiencies. (A) Western blotting analyses of EV markers on BMSCs and BMSC‐derived EVs. Calnexin was used as a negative control. (B) NTA of EVs. (C) Representative TEM photograph of EVs. (D) Biotin pulldown assay and protein identification of biotin‐labelled OBP membrane proteins and EVs. (E) Western blotting analyses of Fn1 on control EVs and FN1‐overexpressed EVs. GAPDH was used as a negative control. The values below the band represent the ratio of the grey value of target proteins and GAPDH by ImageJ. (F) NTA of FN1‐overexpressed EVs. (G) Representative TEM photograph of FN1‐overexpressed EVs. (H) Representative fluorescence photographs of OBPs treated with control EVs or FN1‐overexpressed EVs with DiL label. (I) Quantitative analyses of relative intake of EVs in OBPs. (J) Representative fluorescence photographs of OBPs treated with FITC‐labelled ant‐200b or EVs‐200b. (K) Quantitative analyses of relative intake of ant‐200b in OBPs. (L) Representative fluorescence photographs of OBPs treated with FITC‐labelled ant‐130b or EVs‐130b. (M) Quantitative analyses of relative intake of ant‐130b in OBPs. (N) Representative fluorescence photographs of the femoral heads in nude mice treated with paraperiosteal injection of FITC‐labelled ant‐200b or EVs‐200b. (O) Quantitative analyses of relative intake of ant‐200b in vivo. (P) Representative fluorescence photographs of the femoral heads in nude mice treated with paraperiosteal injection of FITC‐labelled ant‐130b or EVs‐130b. (Q) Quantitative analyses of relative intake of ant‐130b in vivo. (I, K and M) *n* = 3, three fields per sample were selected. Values are shown as mean ± SD. ****p < 0.0001, Student's *t* test. (O, Q) *n* = 5, three fields per sample were selected. Values are shown as mean ± SD. *p < 0.05, ***p < 0.001, Student's *t* test. (C, G) Scale bar = 200 nm. (H, J and L) Scale bar = 50 μm. (N, P) Scale bar = 100 μm. BMSC, bone mesenchymal stem cell; EV, extracellular vesicle; OBP, osteoblast precursor.

The protein detection of recognized EV markers, NTA assays and TEM was performed to identify BMSC‐derived EVs. The EVs highly expressed the EV markers, CD9, CD81 and TSG 101, and weakly expressed the negative control marker Calnexin (Figure 4A). NTA assays showed that the EVs were mainly distributed in 50–120 nm (Figure 4B), and it was observed that under TEM, the EVs presented a spherical shape (Figure 4C). Importantly, the results of biotin pulldown assays and Shotgun proteomics showed that Fn1 on the surface of EVs could interact with the membrane proteins of OBPs (Figure 4D). Accordingly, Fn1 was overexpressed in BMSCs, and the overexpression efficiency of Fn1 in EVs was verified by western blotting (Figure 4E). NTA and TEM assays showed that Fn1‐overexpressed EVs (Fn1‐EVs) remained a spherical shape in a diameter of 50–120 nm (Figure 4F,G).

Before incubation of EVs with OBPs, we stained EVs and Fn1‐EVs with the red fluorescent dye, DiL, to evaluate the intake of EVs by OBPs. It was observed that DiL‐labelled Fn1‐EVs were more absorbed by OBPs than DiL‐labelled control EVs (Figure 4H,I). Then, ant‐200b and ant‐130b were transfected into Fn1‐EVs to prepare ant‐200b‐overexpressed EVs (EVs‐200b) and ant‐130b‐overexpressed EVs (EVs‐130b). EVs‐200b and EVs‐130b showed higher transfection efficiencies than the direct import of ant‐200b and ant‐130b in OBPs in the fluorescence photographs. It could be observed that higher fluorescence intensity of FITC existed in both EVs‐200b and EVs‐130b groups in vitro (Figure 4J–M). In vivo assays showed that the two EVs‐treated OBPs in bone tissue (Runx2 as the marker) have stronger FITC fluorescence (Figures 4N–Q and S3A,B), which indicates that EVs‐200b and EVs‐130b are also more effective in in vivo uptake.

**FIGURE 5:**
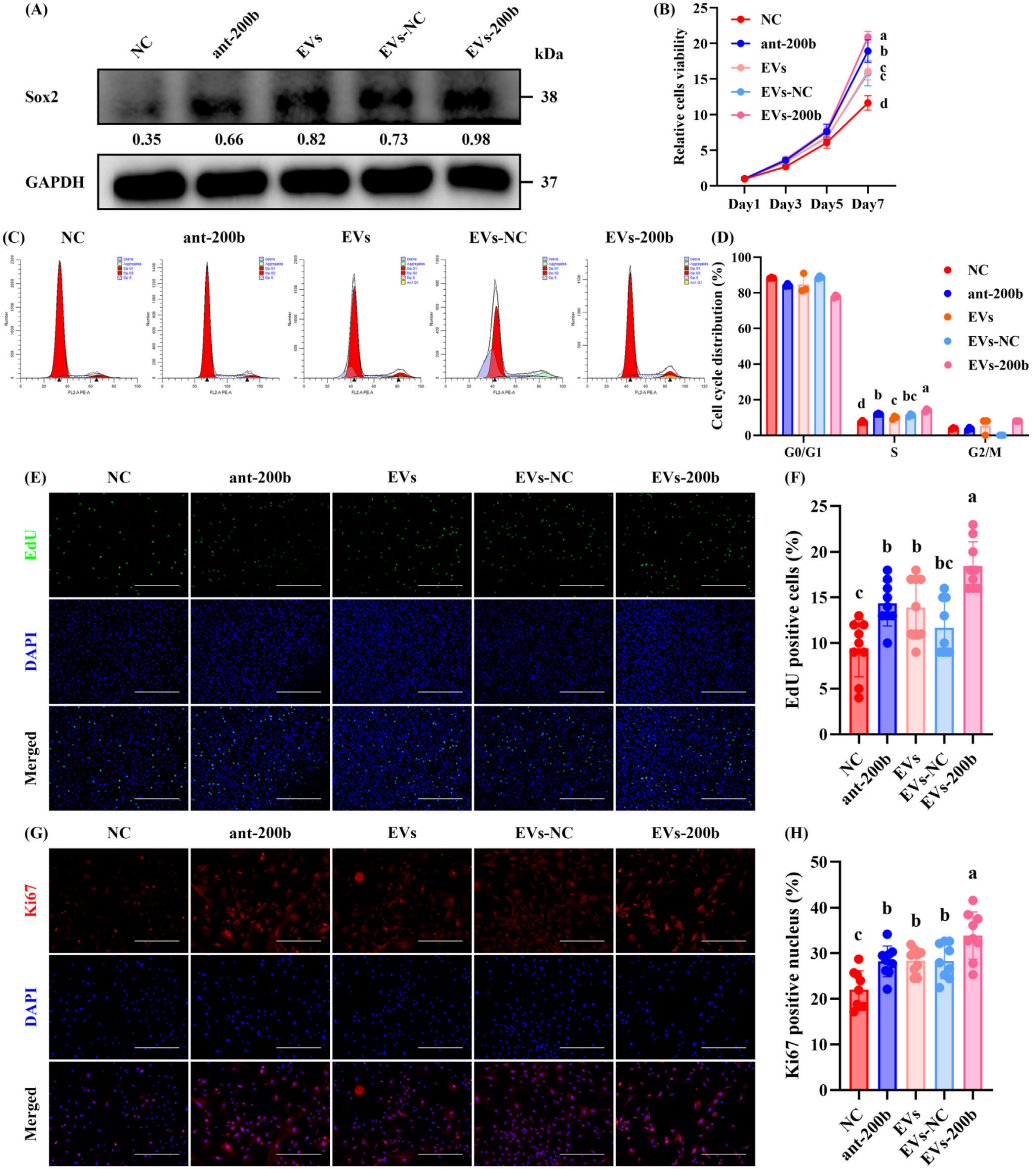
Encapsulation of ant‐200b in OBP‐targeting EVs enhanced its effects on promoting OBP proliferation. (A) Western blotting of Sox2 level in the NC, ant‐200b, EVs, EVs‐NC and EVs‐200b groups. GAPDH was used as a loading control. The values below the band represent the ratio of the grey value of Sox2 and GAPDH by ImageJ. (B) CCK8 assay for cell proliferation of each group. (C) FCM for the cell cycle of each group. (D) Quantitative analyses of OBPs in the G0/G1 phase, S phase and G2/M phase among each group. (E) Representative EdU staining of each group. (F) Quantitative analyses of EdU‐positive cells. (G) Representative IF staining for Ki67 of each group. (H) Quantitative analyses of Ki67‐positive cells. (B) *n* = 3. Values are shown as mean ± SD, two‐way ANOVA. (D) *n* = 3. Values are shown as mean ± SD. ^Letter^ *p* <0.05, one‐way ANOVA. (F, H) *n* = 3, three fields per sample were selected. Values are shown as mean ± SD. ^Letter^ *p* <0.05, one‐way ANOVA. (E) Scale bar = 200 μm. (G) Scale bar = 100 μm. ANOVA, analysis of variance; ant‐NC, antagonist of empty carrier; BMSC, bone mesenchymal stem cell; EV, extracellular vesicle; NC, negative control; OBP, osteoblast precursor.

Then, EVs‐200b and EVs‐130b were used to treat P5 OBPs. Western blotting showed that the EVs‐200b group showed the highest expression of Sox2 protein among each group (Figure 5A). Consistently, P5 OBPs of the EVs‐200b group showed the most obvious proliferation presented as the strongest cell viability (Figure 7B), the highest percentage of cells in S phase (Figure 5C,D), and more EdU‐positive cells (Figure 5E,F). IF training also showed that Ki67 fluorescence intensity was the highest in the EVs‐200b group (Figure 5G,H). It could be observed that the performance of no‐load EVs was significantly weaker than that of EVs‐200b (Figure 5B–H), which proves that the addition of ant‐200b improves EVs in OBP proliferation. Although the direct intervention of ant‐200b was stronger than the NC group in all proliferative parameters, its efficacies were not more obvious than those of EVs and EVs‐NC groups (Figure 5B–H), which verifies the enhanced effect of EVs encapsulation on ant‐200b administration.

**FIGURE 6:**
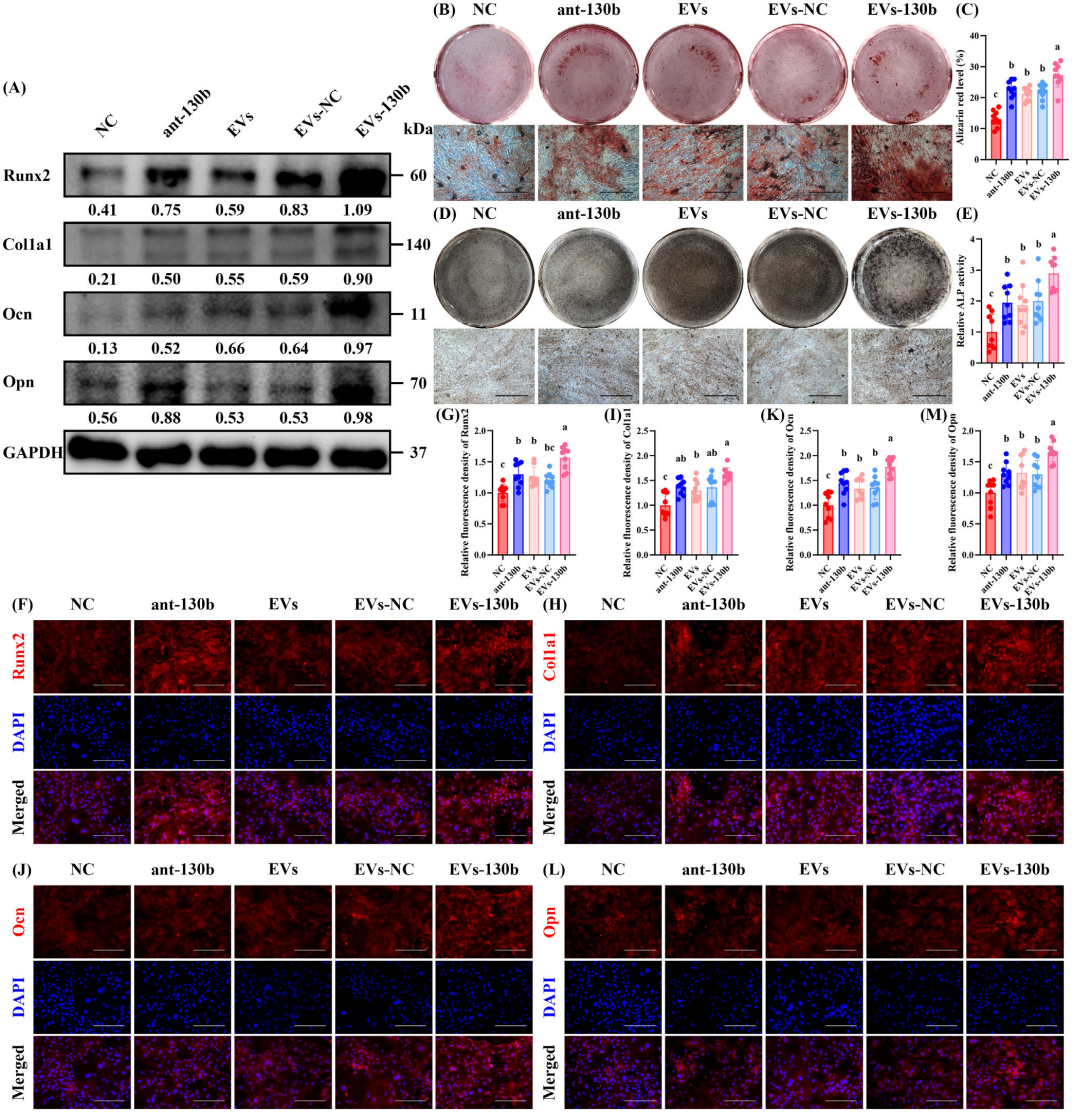
Encapsulation of ant‐130b in OBP‐targeting EVs enhanced its effects on promoting osteogenesis. (A) Western blotting of Runx2, Col1a1, Ocn and Opn levels in the NC, ant‐130b, EVs, EVs‐NC and EVs‐130b groups. GAPDH was used as a loading control. The values below the band represent the ratio of the grey value of target proteins and GAPDH by ImageJ. (B) Representative ARS staining of each group. (C) Quantitative analyses of ARS level. (D) Representative ALP staining of each group. (E) Quantitative analyses of ALP activity. (F–M) Representative IF staining for Runx2 (F), Col1a1 (H), Ocn (J) and Opn (L) of each group, and quantitative analyses of Runx2 (G), Col1a1 (I), Ocn (K) and Opn (M)‐positive cells. (C, E, G, I, K and M) *n* = 3, three fields per sample were selected. Values are shown as mean ± SD. ^Letter^ *p* <0.05, one‐way ANOVA. (B, D) Scale bar = 200 μm. (F, H, J and L) Scale bar = 50 μm. ANOVA, analysis of variance; ant‐NC, antagonist of empty carrier; EV, extracellular vesicle; NC, negative control; OBP, osteoblast precursor.

Also, we found that the protein levels of Runx2, Col1a1, Ocn and Opn were the highest in the EVs‐130b group (Figure 6A). Furthermore, ARS staining and ALP staining showed that EVs‐130b group had the highest levels of calcification and ALP activity, which indicates the strongest osteogenic capacity (Figure 6B–E). As shown in the IF staining, a significant increase in the fluorescence intensity of Runx2, Col1a1, Ocn and Opn was also observed in the EVs‐130b group compared to the ant‐200b, EVs or EVs‐NC group (Figure 6F–M). In addition, the performance of no‐load EVs was obviously weaker than that of EVs‐130b (Figure 6A–M), which proves that the addition of ant‐130b improves EVs in OBP differentiation. Although the direct intervention of ant‐130b was stronger than the NC group in all osteogenic parameters, its efficacies were not more obvious than those of EVs and EVs‐NC groups (Figure 6A–M), which confirms the promoting effect of EVs encapsulation on ant‐130b administration.

### 3.4 Hierarchically injectable hydrogel sequentially delivered EVs‐200b and EVs‐130b in vivo

**FIGURE 7:**
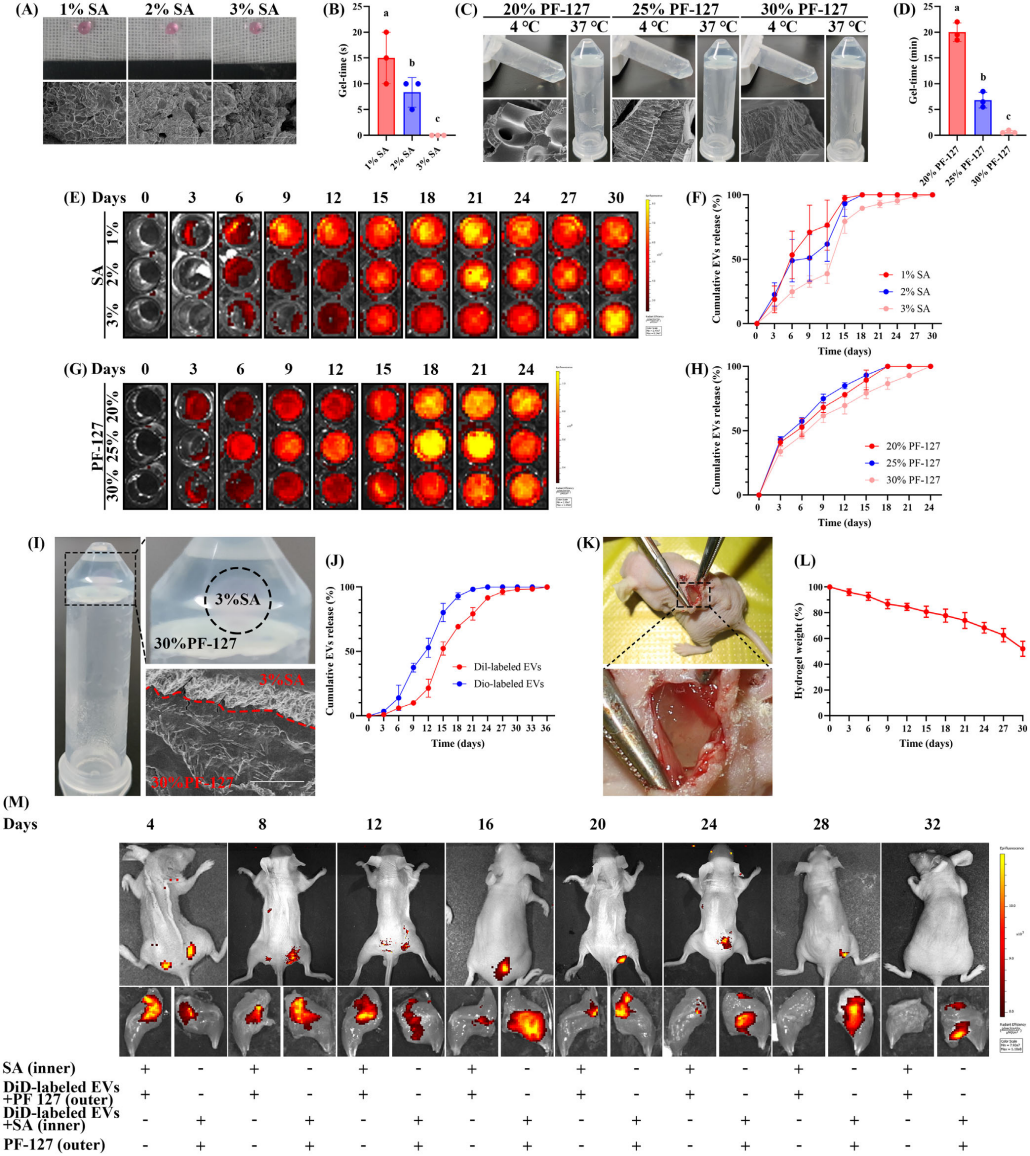
Characterization of hierarchically injectable SA‐PF‐127 hydrogel. (A) Representative macro photographs and TEM photographs of 1%, 2% and 3% SA hydrogel. (B) Quantitative analyses regarding gel formation time of different concentrations of SA hydrogel. (C) Representative macro photographs and TEM photographs of 20%, 25% and 30% PF‐127 hydrogel. (D) Quantitative analyses regarding gel formation time of different concentrations of PF‐127 hydrogel. (E, F) Representative bioluminescence images (E) and quantitative analyses (F) of in vitro EV release of different concentrations of SA hydrogel. (G, H) Representative bioluminescence images (G) and quantitative analyses (H) of in vitro EV release of different concentrations of PF‐127 hydrogel. (I) Representative macro photographs and TEM photographs of the hierarchically injectable hydrogel. (J) Quantitative analyses of in vitro release of dual EVs in the hierarchically injectable hydrogel. (K, L) Representative macro photograph (K) and quantitative analyses (L) regarding in vivo degradation study of the hierarchically injectable hydrogel. (M) In vivo Bioluminescence imaging analyses of hierarchically injectable hydrogel loading DiD‐labelled EVs. (B, D) *n* = 3. Values are shown as mean ± SD. ^Letter^ *p* <0.05, one‐way ANOVA. (A) Scale bar = 100 μm. (C, D and I) Scale bar = 30 μm. ANOVA, analysis of variance; EV, extracellular vesicle; Gel‐time, gel formation time.

The respective role of EVs‐200b or EVs‐130b in OBP proliferation or osteogenesis was known. Accordingly, we experimentally prepared a new biological hydrogel, which leads to the in vivo controlled release of EVs‐200b and EVs‐130b sequentially by hierarchical injection. The designed hydrogel contains SA in the inner layer and PF‐127 in the outer layer. The characteristics of SA and PF‐127 at different concentrations were examined. We found that 3% SA gelled immediately after preparation, while 1% and 2% SA took about 12 and 7 s to form a gel, respectively (Figure 7A,B). The pore diameter of hydrogel decreased with increased concentration (Figure 7A), and the release sustainability of DiD‐labelled EVs mixed in 3% SA was obviously superior to those of the other two concentrations of SA (Figure 7E,F). Moreover, the gel‐time of 30% PF‐127 is significantly faster compared to 20% and 25% PF‐127 (Figure 7C,D). Furthermore, 30% PF‐127 had a more compact microstructure (Figure 7C). Notably, 30% PF‐127 showed excellent performance in the sustained release of DiD‐label EVs, while the other two concentrations of PF‐127 had no obvious continuity in the release of DiD‐label EVs (Figure 7G,H). Thus, 3% SA and 30% PF‐127 were selected to prepare hierarchically injectable hydrogel (Figure 7I). The hierarchical hydrogel enabled the sequential controlled release of DiL‐labelled and DiO‐labelled EVs in vitro for up to 36 days (Figure 7J). And the in vivo degradation investigation showed that the hierarchical hydrogel could last for more than 30 days (Figure 7K,L). The SA and PF‐127 were hierarchically distributed following paraperiosteal injection around the hip (Figure S4). The bioluminescence imaging analyses showed that outer PF‐127 hydrogel could continuously release DiD‐labelled EVs for up to 24 days. While inner SA hydrogel began to release DiD‐labelled EVs on Day 8, and continuously release them for more than 32 days (Figure 7M). These results indicated that the hierarchical hydrogel could first release a kind of particles from the outer PF‐127 and then another kind of particles from the inner SA.

### 3.5 Hierarchical hydrogel with EVs‐200b and EVs‐130b attenuated bone loss in vivo

Finally, we investigated the efficacies of continuous release of EVs‐200b and EVs‐130b controlled by hierarchical hydrogel on the ovariectomy (OVX) osteoporotic mice. As shown in Figure S5, 12‐week‐old female mice were first divided into sham group and OVX group. Three months after surgery, OVX mice were randomly divided into control group, Seq EVs group (sequential injection of EVs), Gel group (empty hierarchical hydrogel), Gel+EVs‐200b group (hierarchical hydrogel with EVs‐200b), Gel+EVs‐130b group (hierarchical hydrogel with EVs‐130b) and Gel+EVs‐200b/EVs‐130b group (hierarchical hydrogel with EVs‐200b and EVs‐130b). For the Seq EVs group, EVs‐200b was paraperiosteally injected weekly around the right hip of OVX mice for the first 2 weeks and EVs‐130b was paraperiosteally injected weekly around the right hip of OVX mice for the next 2 weeks. For the Gel group, SA was paraperiosteally injected around the right hip of OVX mice and PF‐127 was injected on the outside of the SA immediately. For the Gel+EVs‐200b group, SA mixed with EVs‐200b was paraperiosteally injected around the right hip of OVX mice and PF‐127 mixed with EVs‐200b was injected on the outside of the SA immediately. For the Gel+EVs‐130b group, SA mixed with EVs‐130b was paraperiosteally injected around the right hip of OVX mice and PF‐127 mixed with EVs‐130b was injected on the outside of the SA immediately. For the Gel+EVs‐200b/EVs‐130b group, SA mixed with EVs‐130b was paraperiosteally injected around the right hip of OVX mice and PF‐127 mixed with EVs‐200b was injected on the outside of the SA immediately.

**FIGURE 8:**
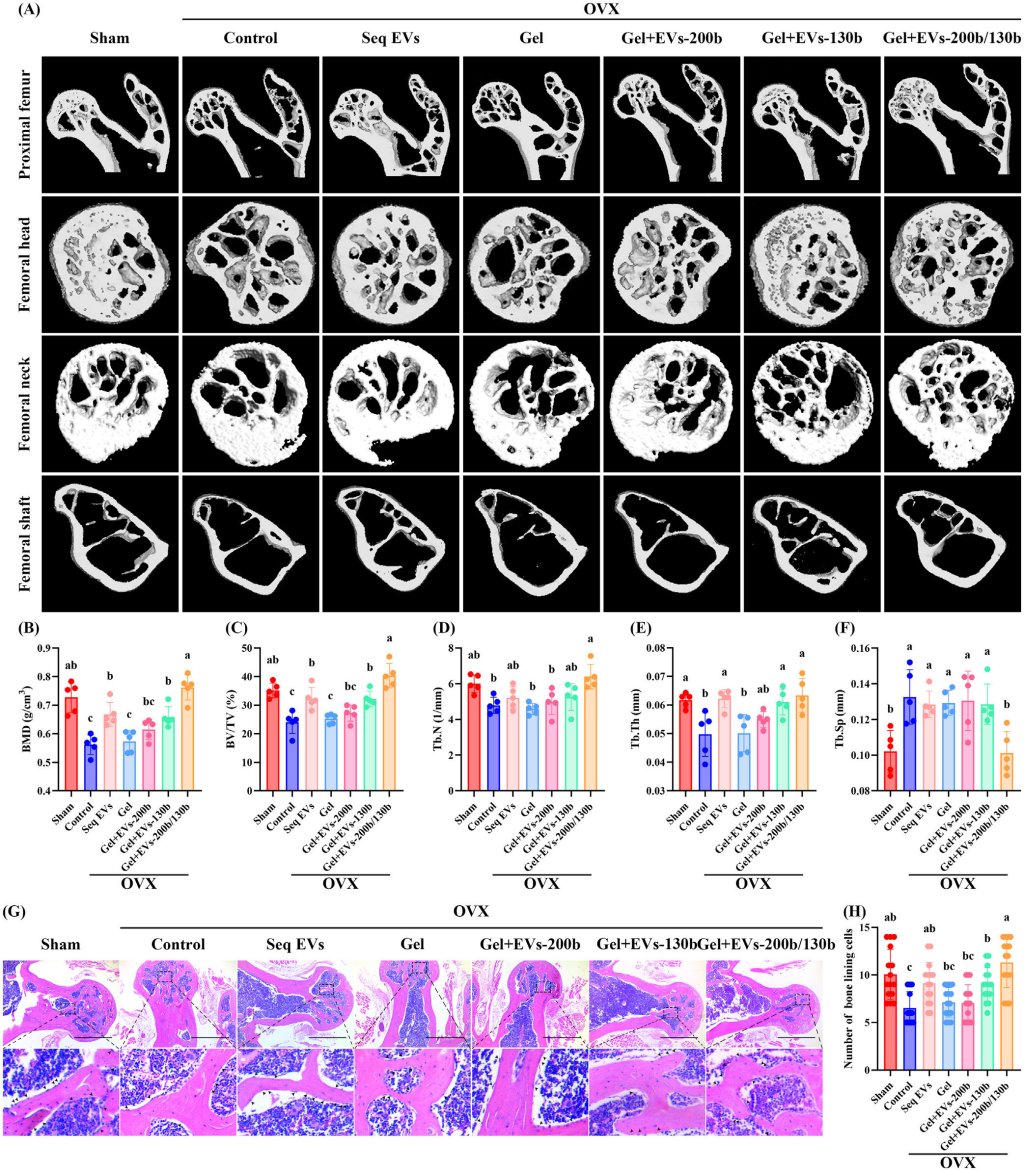
Hierarchical hydrogel with EVs‐200b and EVs‐130b attenuated bone loss. (A) Representative micro‐CT images regarding the three‐dimensional reconstruction of proximal femurs, femoral heads, femoral necks and femoral shafts among Sham mice and control, Seq EVs, Gel, Gel+EVs‐200b, Gel+EVs‐130b and Gel+EVs‐200b/130b groups from OVX mice. (B–F) Quantitative analyses of BMD (B), BV/TV (C), Tb.N (D), Tb.Th (E) and Tb.Sp (F). (G) Representative HE staining of the femoral heads and femoral necks among mice from each group. Arrowhead: bone lining cells. (H) Quantitative analyses of bone lining cells. (B–F) *n* = 5. Values are shown as mean ± SD. ^Letter^ *p* <0.05, one‐way ANOVA. (H) *n* = 5, three fields per sample were selected. Values are shown as mean ± SD. ^Letter^ *p* <0.05, one‐way ANOVA. (G) Scale bar = 25 μm. Sham, mice with sham operation; OVX, ovariectomized mice; Seq EVs, mice receiving the sequential injection of EVs; Gel, mice with empty hierarchical hydrogel; Gel+EVs‐200b, mice with hierarchical hydrogel containing EVs‐200b; Gel+EVs‐130b, mice with hierarchical hydrogel containing EVs‐130b; Gel+EVs‐200b/130b, mice with hierarchical hydrogel containing EVs‐200b and EVs‐130b. ANOVA, analysis of variance; BMD, bone mineral density; EV, extracellular vesicle; Gel‐time, gel formation time.

Micro‐CT and quantitative analyses regarding bone microarchitecture showed that Seq EVs, Gel+EVs‐130b and Gel+EVs‐200b/130b groups all showed an improved bone mass and bone microarchitecture compared to control group, while Gel and Gel+EVs‐200b groups had no effects on all bone parameters. However, Gel+EVs‐200b/130b showed the best efficacy in ameliorating bone loss of OVX mice. It could be observed that treatment with Seq EVs, Gel+EVs‐130b or Gel+EVs‐200b/130b increased BMD, BV/TV and Tb.Th in OVX mice, and Gel+EVs‐200b/130b group had higher Tb.N and lower Tb.Sp (Figure 8A–F). Of note, the Gel+EVs‐200b/130b group displayed higher BMD, BV/TV and lower Tb.Sp than the Seq EVs and Gel+EVs‐130b groups (Figure 8A–F). In addition, H&E and IF staining were performed to evaluate the osteogenic potential of OBPs in vivo. H&E staining showed that the number of bone lining cells was increased in the Seq EVs, Gel+EVs‐130b, and Gel+EVs‐200b/130b groups compared to the control group, and the efficiency of Gel+EVs‐200b/130b group was most obvious (Figure 8G,H).

**FIGURE 9:**
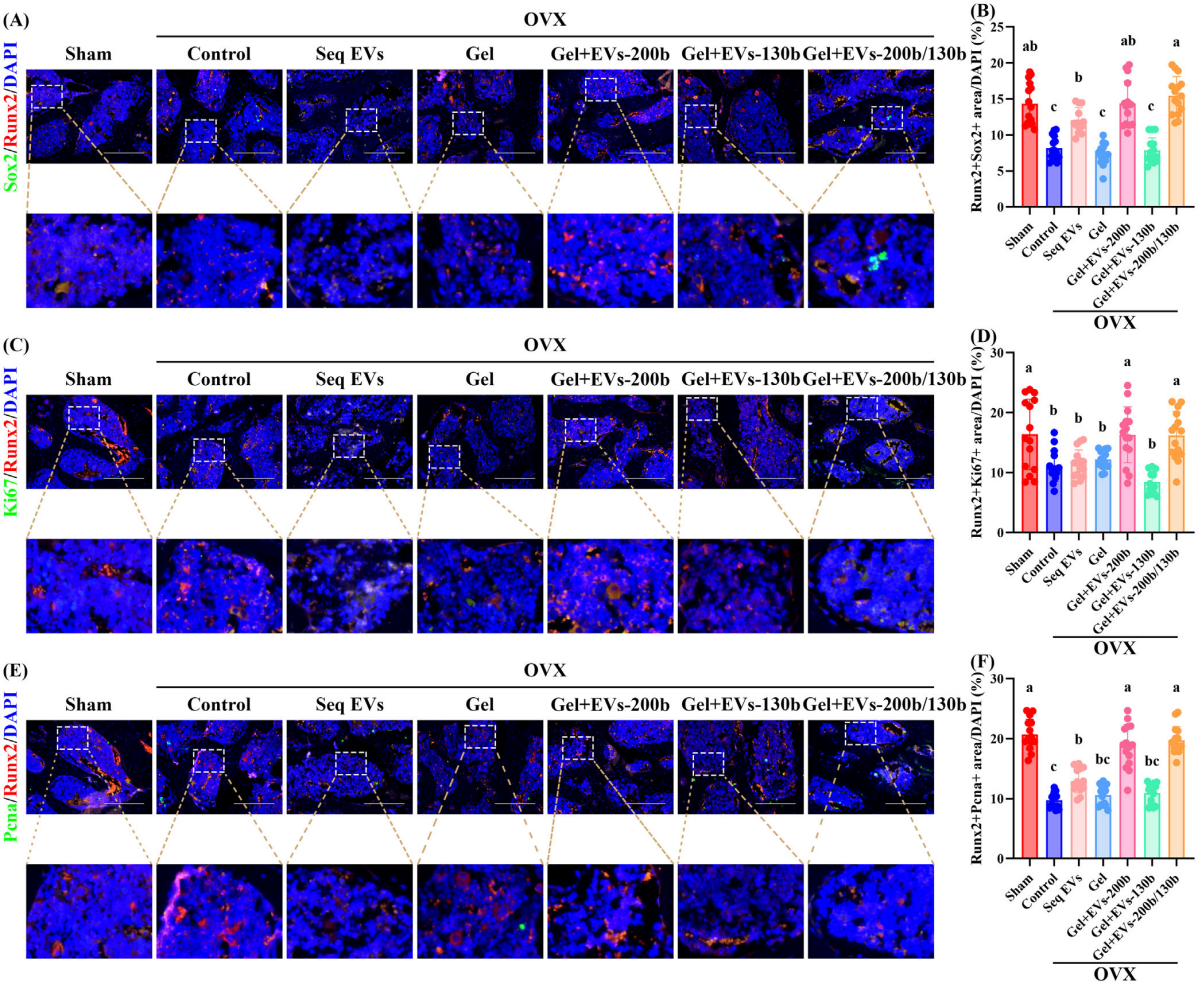
Hierarchical hydrogel with EVs‐200b and EVs‐130b improved the proliferative potential of OBPs in vivo. (A, C and E) Representative IF staining for overlapping fluorescence of Runx2 and Sox2 (A), Ki67 (C) or Pcna (E) in femoral heads among mice from each group. (B, D and F) Quantitative analyses of Runx2 + Sox2‐positive areas (B), Runx2 + Ki67‐positive areas (D) and Runx2 + Pcna‐positive areas (F). (B, D, and F) *n* = 5, three fields per sample were selected. Values are shown as mean ± SD. ^Letter^ *p* <0.05, one‐way ANOVA. (A, C and E) Scale bar = 100 μm. Sham, mice with sham operation; OVX, ovariectomized mice; Seq EVs, mice receiving the sequential injection of EVs; Gel, mice with empty hierarchical hydrogel; Gel+EVs‐200b, mice with hierarchical hydrogel containing EVs‐200b; Gel+EVs‐130b, mice with hierarchical hydrogel containing EVs‐130b; Gel+EVs‐200b/130b, mice with hierarchical hydrogel containing EVs‐200b and EVs‐130b. ANOVA, analysis of variance; EV, extracellular vesicle; Gel‐time, gel formation time.

IF staining of tissues showed that compared with the control group, the fluorescence intensity of Sox2 and Pcna was promoted in the OBPs (Runx2 as the marker) of Seq EVs, Gel+EVs‐200b and Gel+EVs‐200b/130b groups, and Ki67 fluorescence intensity was enhanced in the OBPs of Gel+EVs‐200b and Gel+EVs‐200b/130b groups (Figures 9A–F and S6A–C). Furthermore, the above parameters in Gel+EVs‐200b and Gel+EVs‐200b/130b groups were the highest, which indicated that treatment with Gel+EVs‐200b or Gel+EVs‐200b/130b is the most effective in promoting OBP proliferation in vivo (Figure 9B,D,E).

**FIGURE 10:**
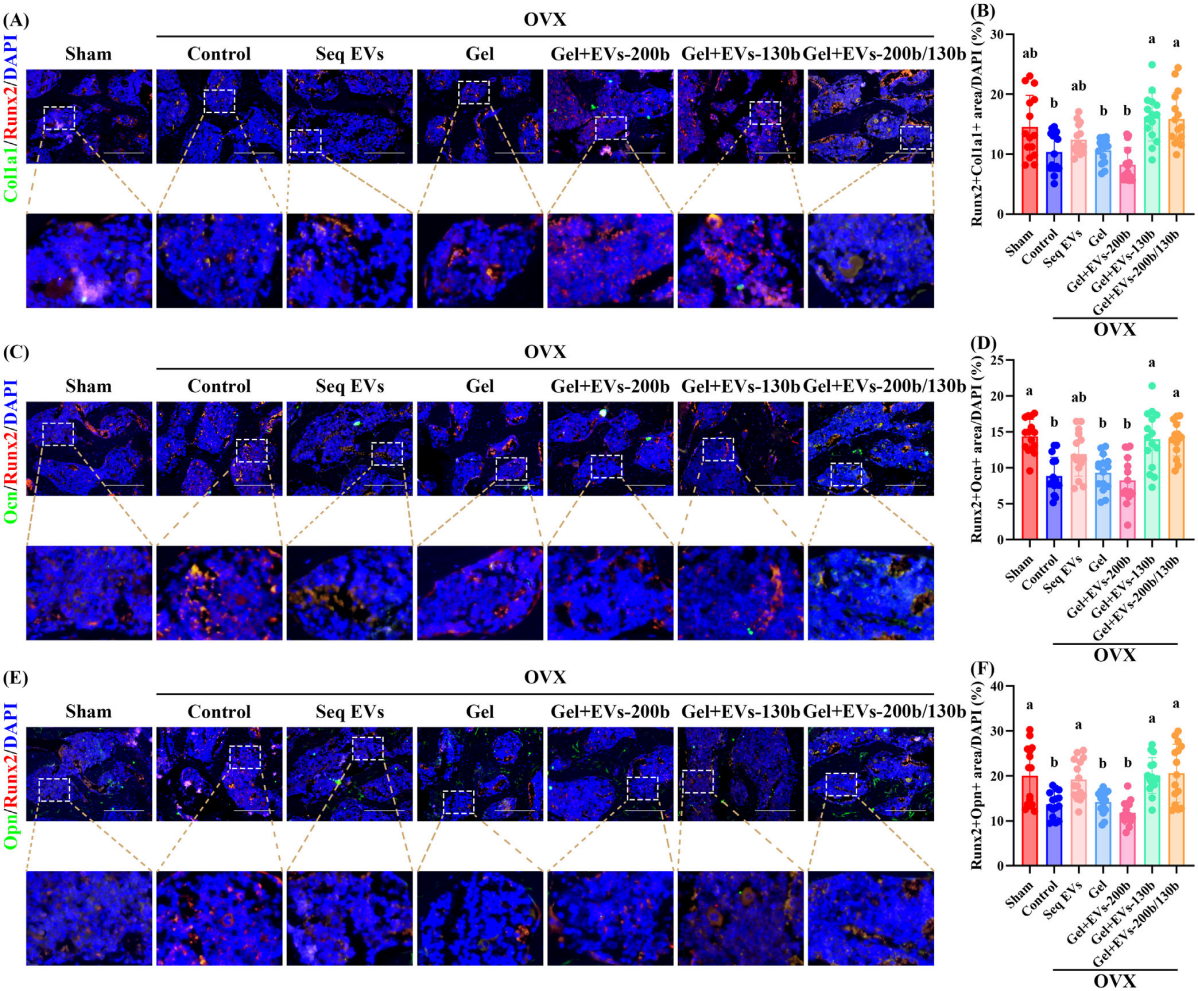
Hierarchical hydrogel with EVs‐200b and EVs‐130b improved the osteogenic potential of OBPs in vivo. (A, C and E) Representative IF staining for overlapping fluorescence of Runx2 and Col1a1 (A), Ocn (C) and Opn (E) in femoral heads among mice from each group. (B, D and F) Quantitative analyses of Runx2 + Col1a1 positive areas (B), Runx2 + Ocn positive areas (D) and Runx2 + Opn positive areas (F). (B, D and F) *n* = 5, three fields per sample were selected. Values are shown as mean ± SD. ^Letter^ *p* <0.05, one‐way ANOVA. (A, C and E) Scale bar = 100 μm. Sham, mice with sham operation; OVX, ovariectomized mice; Seq EVs, mice receiving the sequential injection of EVs; Gel, mice with empty hierarchical hydrogel; Gel+EVs‐200b, mice with hierarchical hydrogel containing EVs‐200b; Gel+EVs‐130b, mice with hierarchical hydrogel containing EVs‐130b; Gel+EVs‐200b/130b, mice with hierarchical hydrogel containing EVs‐200b and EVs‐130b. ANOVA, analysis of variance; EV, extracellular vesicle; Gel‐time, gel formation time; OBP, osteoblast precursor.

In addition, compared with the control group, the fluorescence intensity of Col1a1 and Ocn was enhanced in the OBPs of Gel+EVs‐130b and Gel+EVs‐200b/130b groups, and Opn fluorescence intensity was upregulated in the OBPs of Seq EVs, Gel+EVs‐130b and Gel+EVs‐200b/130b groups (Figures 10A–F and S5A–C). Moreover, the above parameters in Gel+EVs‐130b and Gel+EVs‐200b/130b groups were the highest, which indicated that treatment with Gel+EVs‐130b or Gel+EVs‐200b/130b is the most effective in promoting the osteogenesis of OBPs in vivo (Figures 10A–F and S7A–C). And more Col1a1‐positive and Ocn‐positive OBPs were observed in the Gel+EVs‐130b or Gel+EVs‐200b/130b group compared to the Seq EVs group, both of which had no statistical significance (Figure 10B,D). The Gel+EVs‐200b/130b group did not show statistical differences with the Gel+EVs‐130b group in the above osteogenesis‐related indexes (Figure 10B,D,E). The parameters of OVX mice were all altered compared to Sham mice, which confirms the success of OVX modelling and the effectiveness of our in vivo experimental system.

## 4 DISCUSSION

At present, pathological fracture caused by osteoporosis has become a major social problem because it seriously endangers the health and life of the elderly.^2^ How to effectively treat osteoporotic fractures is of great value for improving people's livelihood. Remarkably, the efficient treatment of the aetiology, namely osteoporosis, is a pivotal strategy for the treatment of such fractures.^5^, ^6^, ^7^, ^8^ The bone cement used in osteoporotic fracture surgery is produced based on the experimental hydrogel. Therefore, the continuous improvement of hydrogel materials in the development phase is of decisive significance for the clinical treatment of such fractures and the recovery of patients. In current, the research of biological hydrogel has become mainstream. Several previous literatures have clarified the positive significance of hydrogels loading with various EVs for bone reconstruction.^10^, ^11^, ^12^, ^13^ However, how to prepare EV‐loaded hydrogels effectively improving BMD locally still has a huge exploration space. The senescence of OBPs is a major reason for the dysregulation of bone formation, which can result in a reduction in OBP proliferation and osteogenic differentiation.^35^, ^36^ Maintaining the above potential of OBPs is the key to improve bone mass loss in the elderly, which is an important subject in the research of osteoporotic fracture. It is known that multiple miRNAs have an obvious role in changing OBP proliferation and osteogenesis due to their targeting effect on the proliferative and osteogenic genes,^24^, ^25^, ^26^, ^28^, ^29^, ^30^, ^31^, ^32^ which leaves an interesting scientific question: whether some antagomiRNAs with osteogenesis‐regulating properties can combine with EV‐loaded hydrogels in treating bone loss. In addition, it is worth noting that the current treatment schemes have defects in maintaining the balance between OBP proliferation and osteogenesis, leading to low efficiency in treating bone loss.^21^, ^22^, ^23^ Therefore, it is urgent to develop a new type of hydrogel based on OBP proliferation and osteogenic differentiation in an orderly manner. Our study developed a revolutionary biological hydrogel, causing the sequential release of the above functional miRNA inhibitors, which solves the previous problems related to osteogenesis and effectively alleviates bone loss in mice in vivo.

In this study, we mined two functional miRNAs through bioinformatics analyses for the first time, miR‐200b‐3p and miR‐130b‐3p, which were verified in vitro. MiR‐200b‐3p was considered to be responsible for the inhibition of OBP proliferation, while miR‐130b‐3p was considered an osteogenic suppressant. The functions of the two miRNAs on OBPs attributed to their respective abilities to target the 3′‐UTR of Sox2 and Runx2 mRNAs. Sox2 serves as a transcription factor in maintaining the self‐renew and proliferation of OBPs,^37^, ^38^ while Runx2 is a key transcription factor that is integrally accountable for osteogenesis, resulting in endochondral and intramembranous ossification.^39^ Accordingly, the application of specific antagonists may release the targeted inhibition of Sox2 and Runx2, thus contributing to OBP proliferation and osteogenesis. As expected, we found that the inhibition of miR‐200b‐3p or miR‐130b‐3p with ant‐200b or ant‐130b could promote the proliferation and osteogenic differentiation of OBPs, respectively. It should be noted that ant‐200b administration also slightly improved the calcification ability of ageing OBPs, which indicates that the enhanced OBP proliferation with ant‐200b provides a more sufficient precursor reserve for subsequent osteogenic differentiation. Then, we constructed a new type of EVs, which contains the overexpressed Fn1, an interacting molecule with the membrane proteins of OBPs. Many current osteoporosis drugs cannot effectively act on the target cells due to their low targeting, which is the main reason for the poor efficacy and obvious side effects in treating various bone loss.^40^, ^41^, ^42^ Previous studies showed that EVs can attach to related cells through the overexpression of specific molecules, which interact with corresponding membrane proteins.^43^, ^44^, ^45^, ^46^ This study clarified that the designed EVs are directionally bound to OBPs because of the overexpression of Fn1, a screened attachment protein. The effectiveness of EVs loaded with specific antagomiRNAs has also been confirmed by in vitro and in vivo assays. It could be observed that the constructed OBP‐targeting EVs more effectively brought ant‐200b and ant‐130b into OBPs and further enhanced the functions of ant‐200b and ant‐130b in promoting the proliferation and osteogenic differentiation of OBPs. Accordingly, we believe that the functional EVs integrating OBP targeting and specific antagomiRNAs can be applied as an important biological basis in the preparation of orthopaedic hydrogel materials. Moreover, this type of EVs with overexpressed Fn1 may also carry other drugs into OBPs more effectively due to the targeted combination with OBPs, which presents a creative significance for the future treatment of osteoporosis.

We designed the paraperiosteal injection scheme of hierarchical hydrogel with PF‐127 (outer layer) and SA (inner layer), which is around the hip joints of OVX osteoporotic mice. Relying on the above scheme, the controlled release of EVs‐200b and EVs‐130b was realized, which enabled EVs‐200b and EVs‐130b to be delivered to OBPs in vivo in turn. As expected, the above scheme effectively improved the in vivo osteogenic capacity of OBPs and bone microarchitecture of the proximal femurs. Structured biomaterials facilitate the sequential release of designed drugs and the biological regulation of target cells. Lee et al. constructed a double cryogel system with gelatin and chitosan for the sequential release of vascular endothelial growth factor (Vegf) and bone morphogenetic protein (Bmp)‐4 to improve bone regeneration.^47^ Zheng et al. constructed a programmed surface composed of poly (lactide‐co‐glycolide) and alendronate (ALN), which was loaded on the nano‐hydroxyapatite in the inner layer and Il‐4 in the outer layer, respectively.^48^ The above design can sequentially regulate the immunomodulatory microenvironment and bone regeneration to ameliorate bone‐implant osseointegration.^48^ Similar results were also presented in other studies.^49^, ^50^, ^51^ In addition, the injectable hydrogel could prevent EVs from rapid clearance and avoid the multiple injections of EVs in vivo.^52^ Here, EVs‐200b and EVs‐130b were loaded into the hierarchically injectable hydrogel to extend the half‐life of the EVs, thereby enhancing the therapeutic effects of corresponding EVs on improving bone mass in vivo. This novel biomaterial is expected to be suitable for the local increase of BMD to prevent osteoporotic fractures in corresponding sites.

There are still some limitations in our study. First, the hierarchically injectable hydrogel was prepared with SA and PF‐127, which were moulded after injection. Therefore, the shape and thickness of these two hydrogels are difficult to accurately control. The above problems can be solved through the research and development of the injectable reagent based on hierarchical nanoparticles due to its promotion of the precise release of drugs.^53^, ^54^, ^55^ Furthermore, the time to observe the effects of the EV‐loaded hydrogel on the bone mass in the OVX mice was 1 month after hydrogel injection, which was much shorter than that of hydrogel degradation in vivo. In the future, how to prolong the action time of this biological hydrogel is an urgent problem to be solved.

## 5 CONCLUSION

In the present study, we constructed OBP‐targeting EVs by overexpressing Fn1 and transfected ant‐200b and ant‐130b into this type of EVs. The efficacy of the above recombinant EVs has been confirmed. Subsequently, we loaded EVs‐200b and EVs‐130b into a hierarchically injectable hydrogel composed of PF‐127 (outer layer) and SA (inner layer), and the novel biological hydrogel can sequentially promote OBP proliferation and osteogenic differentiation to increase bone mass in vivo. Taken together, our study provides an innovative biomaterial with cell targeting, which has significant transforming value in improving bone loss and treating osteoporotic fractures.

## AUTHOR CONTRIBUTIONS

Hanhao Dai, Yunlong Yu and Junyong Han performed the majority of the experiments in the study. Jun Luo, Jiahui Li, Chao Song, Zhibo Deng and Yijing Wu contributed to the analysis of experimental data. Hanhao Dai, Dianshan Ke and Jie Xu contributed to the study design, manuscript writing and provided experimental funding support. All authors read and approved the final manuscript.

## CONFLICT OF INTEREST STATEMENT

The authors declare no conflict of interest.

## Supporting information


**FIGURE S1.** The proliferative potential of OBPs was impaired with ageing.
**FIGURE S2.** The osteogenic potential of OBPs were impaired with ageing.
**FIGURE S3.** Representative fluorescence photographs of the femoral heads in nude mice treated with paraperiosteal injection of FITC‐labelled ant‐200b, EVs‐200b, ant‐130b or EVs‐130b, including single and merged fluorescence.
**FIGURE S4.** The SA and PF‐127 were hierarchically distributed following paraperiosteal injection around the hip.
**FIGURE S5.** Summary diagram of animal experiments.
**FIGURE S6.** Representative IF staining for overlapping fluorescence of Runx2 and Sox2 (A), Ki67 (B) or Pcna (C) in femoral heads among mice from each group, including single and merged fluorescence.
**FIGURE S7.** Representative IF staining for overlapping fluorescence of Runx2 and Col1a1 (A), Ocn (B) or Opn (C) in femoral heads among mice from each group, including single and merged fluorescence.

**DATA S1.** Supporting Information

## Data Availability

The data that support the findings of this study are available from the corresponding author upon reasonable request.
