# Supplementary material for: A novel biologically hierarchical hydrogel with osteoblast precursor‐targeting extracellular vesicles ameliorates bone loss in vivo via the sequential action of antagomiR‐200b‐3p and antagomiR‐130b‐3p
Source: Cell Prolif. 2023 Feb 14;56(8):e13426. doi: 10.1111/cpr.13426 (PMC10392057; doi:10.1111/cpr.13426)
Supplement: Supplementary file 1 — FIGURE S1. The proliferative potential of OBPs was impaired with ageing. FIGURE S2. The osteogenic potential of OBPs were impaired with ageing. FIGURE S3. Representative fluorescence photographs of the femoral heads in nude mice treated with paraperiosteal injection of FITC‐labelled ant‐200b, EVs‐200b, ant‐130b or EVs‐130b, including single and merged fluorescence. FIGURE S4. The SA and PF‐127 were hierarchically distributed following paraperiosteal injection around the hip. FIGURE S5. Summary diagram of animal experiments. FIGURE S6. Representative IF staining for overlapping fluorescence of Runx2 and Sox2 (A), Ki67 (B) or Pcna (C) in femoral heads among mice from each group, including single and merged fluorescence. FIGURE S7. Representative IF staining for overlapping fluorescence of Runx2 and Col1a1 (A), Ocn (B) or Opn (C) in femoral heads among mice from each group, including single and merged fluorescence. [file CPR-56-e13426-s002.docx]

**Supplementary materials**


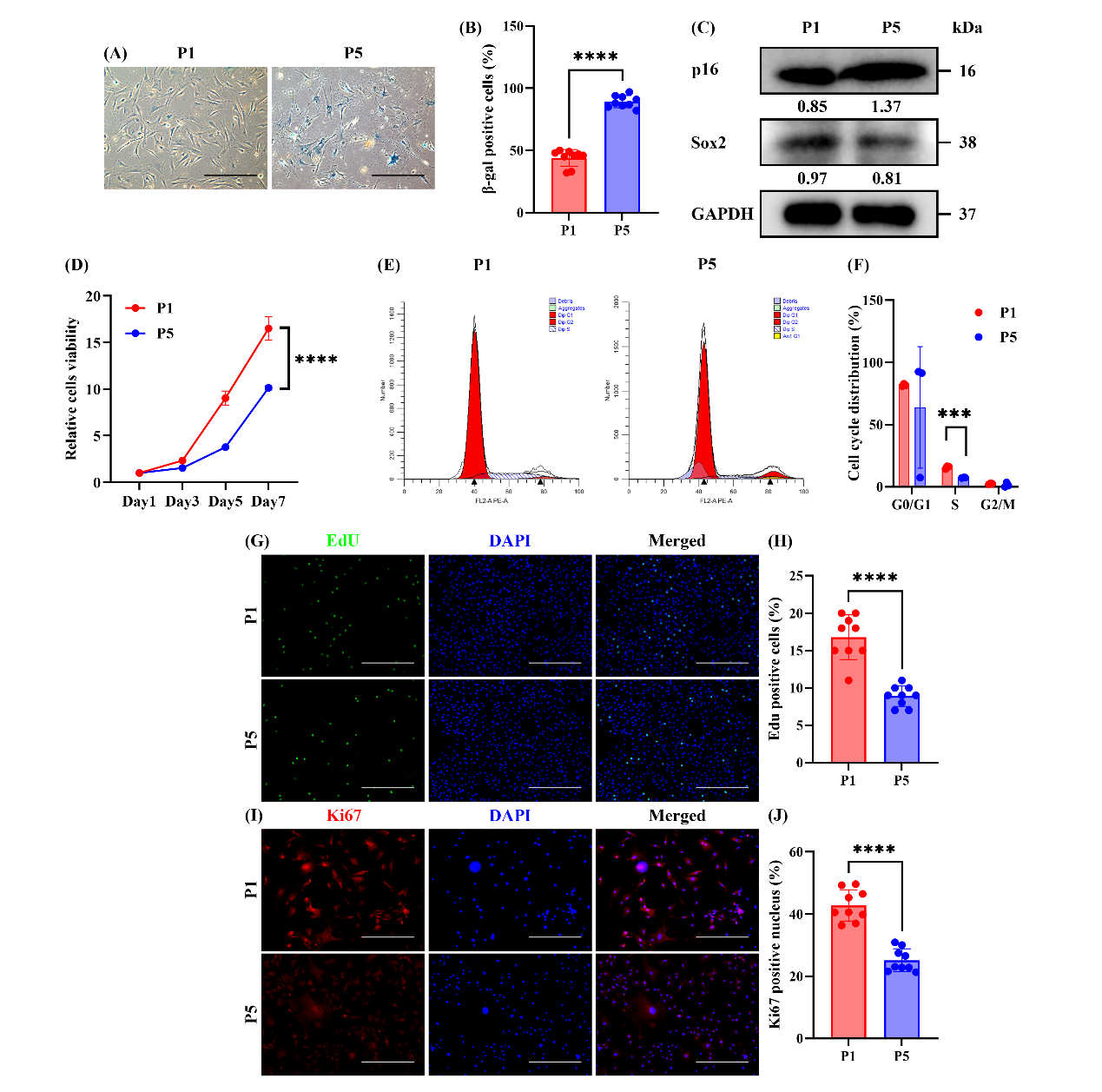
**Fig. S1. The proliferative potential of OBPs was impaired with aging.** (A) Representative β-gal staining of P1 and P5 OBPs. (B) Quantitative analyses of β-gal-positive cells. (C) Western blotting of Sox2 and P16 levels of P1 and P5 OBPs. GAPDH was used as a loading control. The values below the band represent the ratio of the gray value of target proteins and GAPDH by ImageJ. (D) CCK8 assay for cell proliferation of P1 and P5 OBPs. (E) FCM for the cell cycle of P1 and P5 OBPs. (F) Quantitative analyses of P1 and P5 OBPs in the G0/G1 phase, S phase, and G2/M phase. (G) Representative EdU staining of P1 and P5 OBPs. (H) Quantitative analyses of EdU-positive cells. (I) Representative IF staining for Ki67 of P1 and P5 OBPs. (J) Quantitative analyses of Ki67-positive cells. (B, H, and J) n = 3, three fields per sample were selected. Values are shown as mean ± SD. *****P* < 0.0001, student’s t-test. (D) n = 3. Values are shown as mean ± SD. *****P* < 0.0001, two-way ANOVA. (F) n = 3. Values are shown as mean ± SD. ****P* < 0.001, student’s t-test. (A and I) Scale bar = 100 μm. (G) Scale bar = 200 μm. P1, the first generation of OBPs; P5, the fifth generation of OBPs.

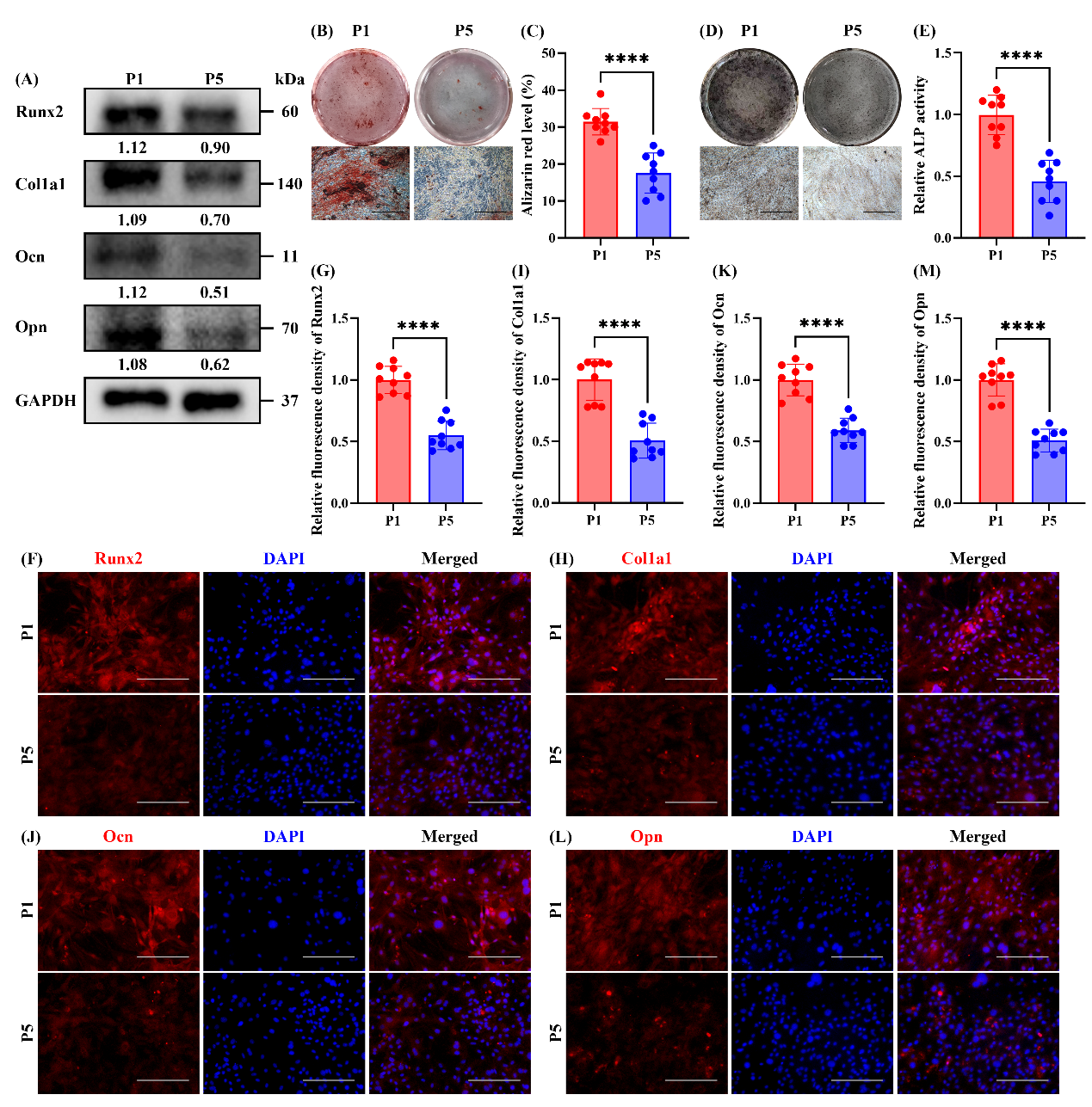
**Fig. S2. The osteogenic potential of OBPs were impaired with aging.** (A) Western blotting of Runx2, Col1a1, Ocn, and Opn levels of P1 and P5 OBPs. GAPDH was used as a loading control. The values below the band represent the ratio of the gray value of target proteins and GAPDH by ImageJ. (B) Representative ARS red staining of P1 and P5 OBPs. (C) Quantitative analyses of ARS level. (D) Representative ALP staining of P1 and P5 OBPs. (E) Quantitative analyses of ALP activity. (F-M) Representative IF staining for Runx2 (F), Col1a1 (H), Ocn (J), and Opn (L) of P1 and P5 OBPs, and quantitative analyses of Runx2 (G), Col1a1 (I), Ocn (K), and Opn (M)-positive cells. (C, E, G, I, K, and M) n = 3, three fields per sample were selected. Values are shown as mean ± SD. ****P < 0.0001, student’s t-test. (B, D) Scale bar = 200 μm. (F, H, J, and L) Scale bar = 50 μm. P1, the first generation of OBPs; P5, the fifth generation of OBPs.

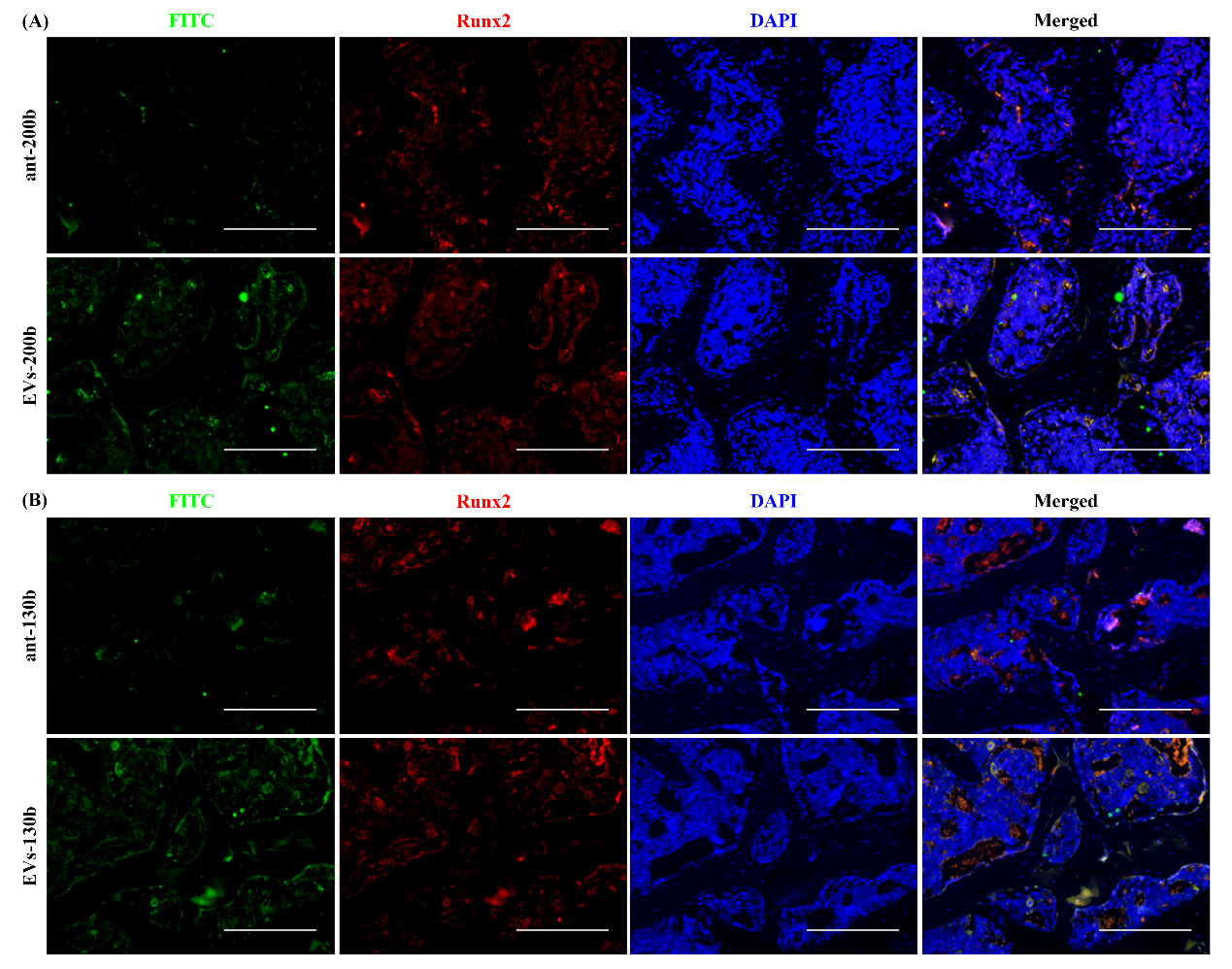
**Fig. S3. Representative fluorescence photographs of the femoral heads in nude mice treated with paraperiosteal injection of FITC-labeled ant-200b, EVs-200b, ant-130b, or EVs-130b, including single and merged fluorescence.**

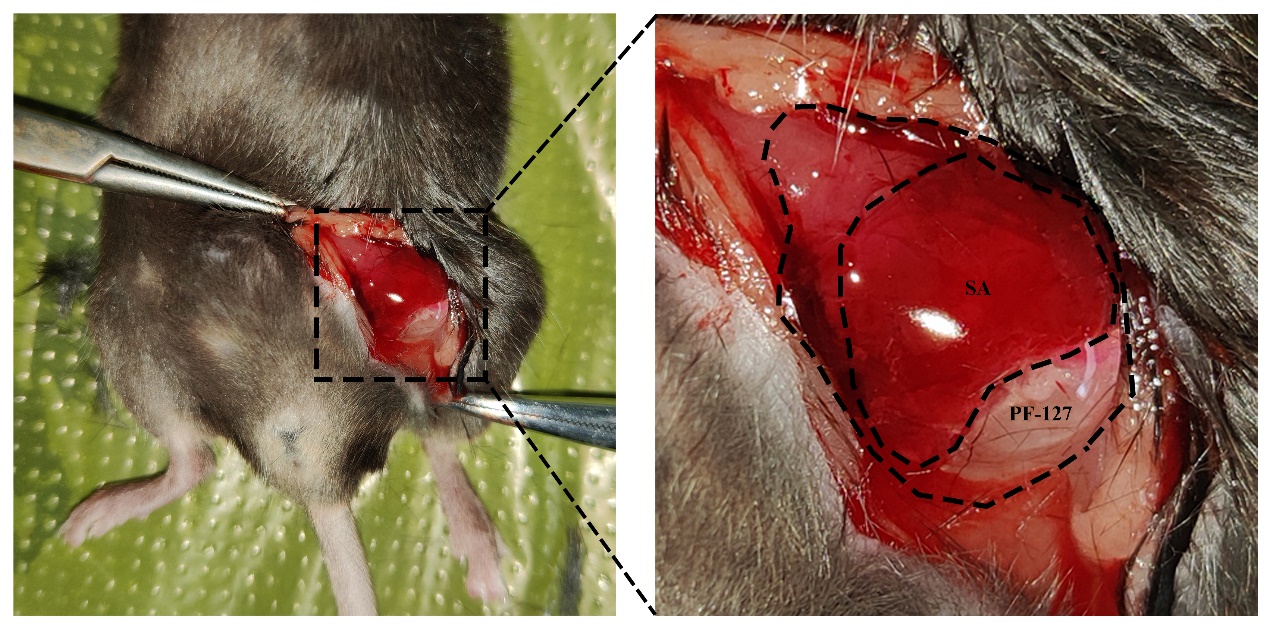


**Fig. S4. The SA and PF-127 were hierarchically distributed following paraperiosteal injection around the hip.**

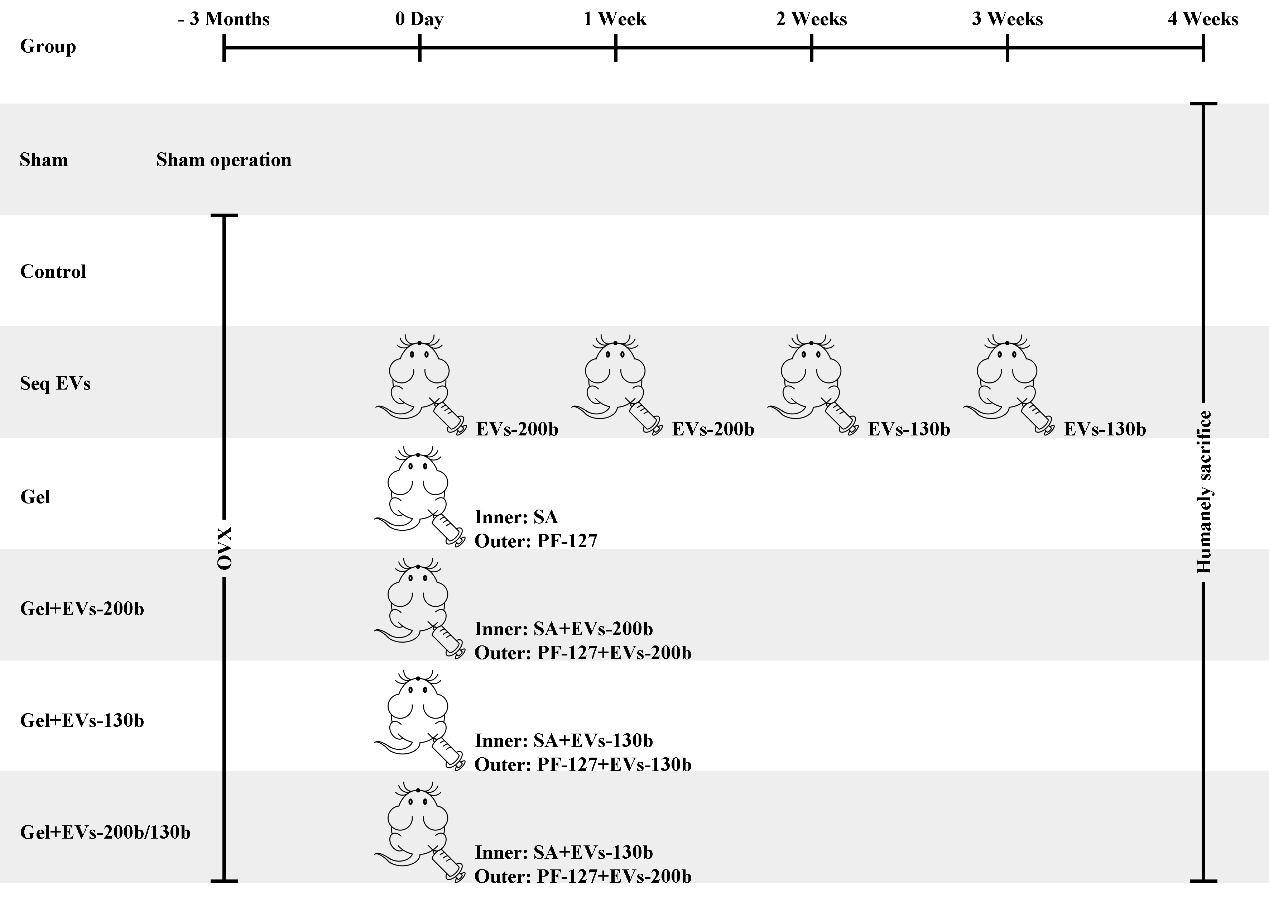


**Fig. S5. Summary diagram of animal experiments.** 12-week-old female mice were firstly divided into sham group and OVX group. 3 months after surgery, OVX mice were randomly divided into control group, Seq EVs group, Gel group, Gel+EVs-200b group, Gel+EVs-130b group, Gel+EVs-200b/EVs-130b group. For the Seq EVs group, EVs-200b was paraperiosteally injected weekly around the right hip of OVX mice for the first 2 weeks and EVs-130b was paraperiosteally injected weekly around the right hip of OVX mice for the next 2 weeks. For the Gel group, SA was paraperiosteally injected around the right hip of OVX mice and PF-127 was injected on the outside of the SA immediately. For the Gel+EVs-200b group, SA mixed with EVs-200b was paraperiosteally injected around the right hip of OVX mice and PF-127 mixed with EVs-200b was injected on the outside of the SA immediately. For the Gel+EVs-130b group, SA mixed with EVs-130b was paraperiosteally injected around the right hip of OVX mice and PF-127 mixed with EVs-130b was injected on the outside of the SA immediately. For the Gel+EVs-200b/EVs-130b group, SA mixed with EVs-130b was paraperiosteally injected around the right hip of OVX mice and PF-127 mixed with EVs-200b was injected on the outside of the SA immediately.

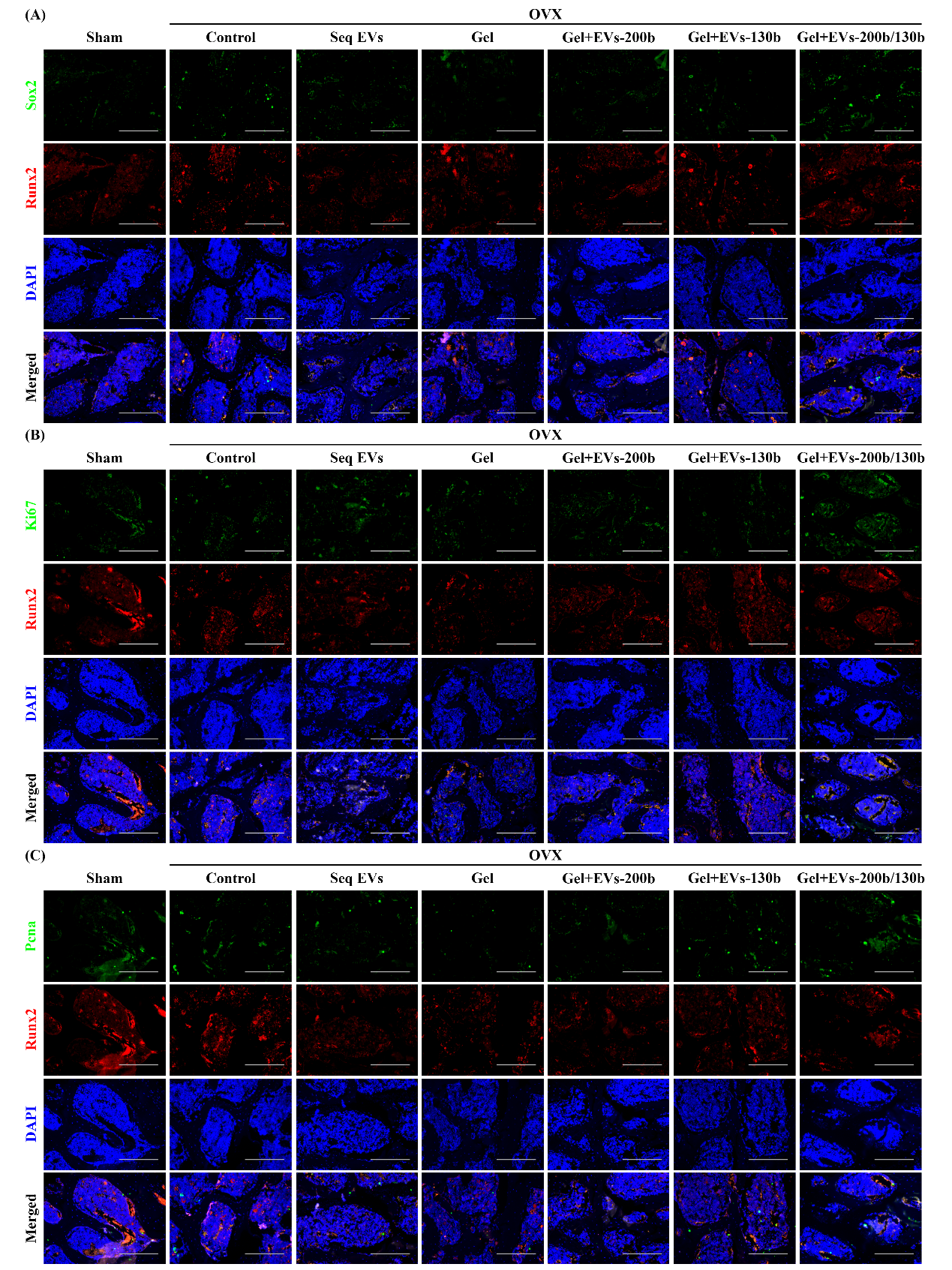


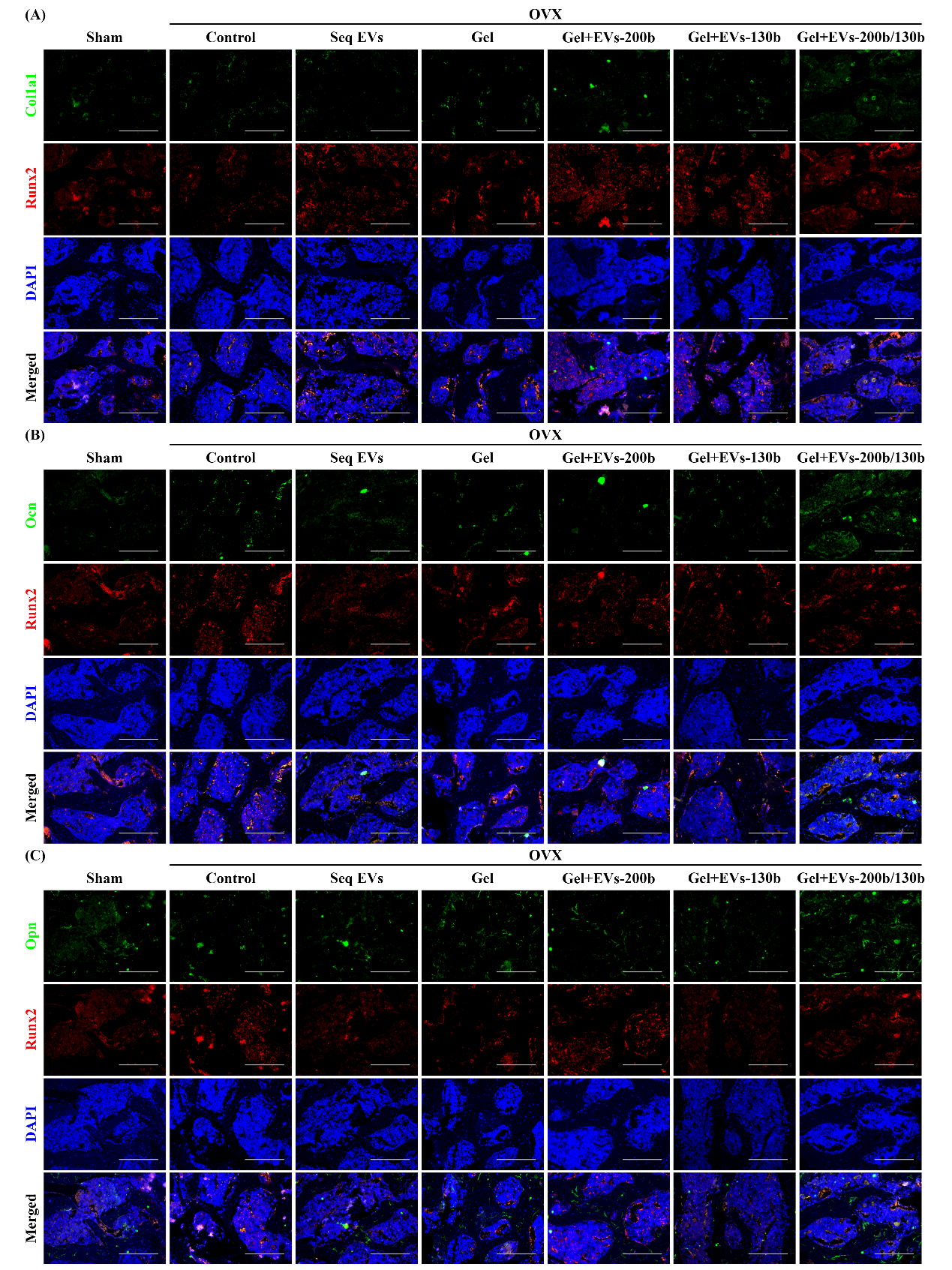
**Fig. S6. Representative IF staining for overlapping fluorescence of Runx2 and Sox2** (A)**, Ki67** (B)**, or Pcna** (C) **in femoral heads among mice from each group, including single and merged fluorescence.**
**Fig. S7. Representative IF staining for overlapping fluorescence of Runx2 and Col1a1** (A)**, Ocn** (B)**, or Opn** (C) **in femoral heads among mice from each group, including single and merged fluorescence.**
