# Supplementary material for: A novel biologically hierarchical hydrogel with osteoblast precursor‐targeting extracellular vesicles ameliorates bone loss in vivo via the sequential action of antagomiR‐200b‐3p and antagomiR‐130b‐3p
Source: Cell Prolif. 2023 Feb 14;56(8):e13426. doi: 10.1111/cpr.13426 (PMC10392057; doi:10.1111/cpr.13426)
Supplement: Supplementary file 2 — DATA S1. Supporting Information [file CPR-56-e13426-s001.docx]

**Supplementary materials and methods**

**Animals**

10 3-day-old neonatal male C57/BL6 mice, 10 4-week-old male C57/BL6 mice, 35 12-week-old female C57/BL6 mice, and 30 8-week-old male nude mice were provided by the Animal Experiment Center of Fujian Medical University. All animal experiments were approved by the Animal Ethics Committee of Fujian Medical University (NO. 2019-0134). And all animal experiments were carried out in accordance with the ARRIVE guidelines and the National Research Council's Guide for the Care and Use of Laboratory Animals.

**Isolation of primary OBPs and BMSCs**

OBPs were isolated as described previously [1]. In brief, the 3-day-old neonatal mice were sacrificed and disinfected by using 75 % ethyl alcohol. Calvariae were harvested and washed with PBS supplemented with 1 % penicillin/streptomycin twice. Then calvariae were minced and digested with 2 mg/ml collagenase type II dissolved in serum-free dulbecco's modified eagle medium (DMEM) at 37 ℃ with continuous shaking. Keep changing fresh 2 mg/ml collagenase type II every 20 min for 5 times. After digestion, the digestion solution was filtered by using a 70 μm nylon mesh cell strainer (431751, Corning, USA). The cells were centrifuged and resuspended in high glucose DMEM medium supplemented with 10 % FBS and 1 % penicillin/streptomycin and cultured at 37 ℃ in 5 % CO2.

To isolate BMSC, 4-week-old mice were sacrificed and disinfected by using 75 % ethyl alcohol. The long bones of hind limbs were harvested. BMSCs were isolated by flushing the bone marrow from the long bones of hind limbs [23]. Then BMSCs were cultured in α-Minimum Essential Medium (α-MEM) supplemented with 10 % FBS and 1 % penicillin/streptomycin at 37 ℃ in 5 % CO2.

Human embryonic kidney 293T (293T) cells (TCH-C101, Cas9XTM, China) were purchased from Cas9XTM Biotechnology and incubated in 293T growth medium (TCH-G101, Cas9XTM, China) at 37 ℃ in 5 % CO2.

**Senescence-associated β-galactosidase (β-gal) staining**

Senescence-associated β-gal staining was performed using a senescence β-gal staining kit (C0602, Beyotime, China) according to the manufacturer’s instructions. Briefly, the adherent cells were fixed in 4 % paraformaldehyde and stained with β-gal staining solution at room temperature (RT) overnight.

**Osteogenic differentiation assay**

Osteogenic differentiation assay was performed using an osteogenic induction medium (MUXMT-90021, cyagen, China) according to the manufacturer’s instructions. In brief, OBPs were plated in 24-well culture plates at a density of 10000 cells per well and cultured with high glucose DMEM medium supplemented with 10 % FBS and 1 % penicillin/streptomycin. After OBPs reached confluence, the medium was changed to the osteogenic induction medium. The medium was changed every 3 days. After 7-day incubation, alkaline phosphatase (ALP) staining was performed by using an ALP stain kit (G1481, Solarbio, China) according to the manufacturer’s instructions. And after 21-day incubation, alizarin red staining (ARS) was performed by using alizarin red solution (ALIR-10001, cyagen, China) according to the manufacturer’s instructions.

**Cell counting kit-8 (CCK-8) assay**

Cell proliferation was assayed by CCK-8 staining (C0038, Dojindo, Japan). 1000 OBPs were seeded in 96-well plates. 10 μL CCK-8 solution was added and incubated for 1h at 37 ℃. The absorbance was determined by a microplate reader (SpectraMax iD5, MD, USA). The CCK-8 assay was performed on day1, 3, 5, and 7 after OBP seeding. These experiments were repeated three times.

**EdU proliferation assay**

According to the manufacturer's instructions (KGA331-100, Keygen Biotech, China), 10 μM EDU medium was prepared. The OBPs were seeded into the 96-well plate. After the OBPs reached 80% confluence, the medium was changed to 100 μL of EDU medium and cultured at 37 ℃ in 5 % CO2 for 2 h. Then the OBPs were fixed and permeable. Subsequently, OBPs were stained with Click-iT staining solution for 30 min at RT and counterstained with 4’, 6-diamidino-2-phenylindole (DAPI) (0100-20, SouthernBiotech, USA). Finally, the OBPs were observed under the fluorescence microscope.

**Flow cytometric analysis (FCM)**

FCM for the cell cycle of OBPs was performed by using a cell cycle detection kit (KGA512, Keygen Biotech, China) according to the manufacturer’s instructions. Briefly, OBPs were harvested by trypsinization. Then, OBPs were washed once with PBS. Subsequently, OBPs were centrifugation and fixed in 70% cold ethanol at -20 °C for 2 h. Fixed OBPs were washed twice with PBS. Next, OBPs were stained with propidium iodide (PI)/RNase A solution for 60 min at RT in the dark. Flow cytometry analysis was performed on a FACS verse (Accuri C6 Plus, BD, USA).

**Bioinformatics analysis**

The GSE57127 microarray data were obtained from the GEO. Differentially expressed genes (DEGs) were identified by using the R package “limma” [2]. The cutoff criteria were |log2 fold change (FC)|> 0.5 and adjusted P < 0.05. The heatmaps were formed by using the R package “pheatmap” [3]. The predicted miRNA-mRNA interaction data were collected from the miRWalk 3.0 database (http://mirwalk.umm.uni-heidelberg.de/), TargetScan database (https://www.targetscan.org/mamm_31/), and miRDB database (http://www.mirdb.org/).

**Quantitative real-time PCR (qRT-PCR)**

Total RNAs from SCs were extracted by using TRIzol. Bulge-LoopTM miRNA qRT-PCR Starter Kit (R11067.3, Ribobio, China) was used to generate cDNA according to the manufacturer’s instructions. And Bulge-LoopTM miRNA qRT-PCR Primer (R10031.8, Ribobio, China) was used to perform qRT-RCP, and U6 small nuclear RNA was used as an internal control. Gene expression was quantified by using the 2−ΔΔCt method. These experiments were repeated three times. The primer sequences are shown as follows:

miR-129-5p: F: 5’-CTTTTTGCGGTCTGGGCTTGC-3’; R: 5’-AGTGCAGGGTCCGAGGTATT-3’, miR-200b-3p: F: 5’-TAATACTGCCTGGTAATGATG-3’, R: 5’-CTCAACTGGTGTCGTGGA-3’; miR-1896: F: 5’-AAGGCTTTCCCACACTCATTACAG-3’, R: 5’-CTCAACTGGTGTCGTGGA-3’; miR-130b-3p: F: 5’-CAGTCCACCAGTGCAATGATG-3’, R: 5’-TATGCTTGTTCTCGTCTCTGTGTC-3’; miR-17-3p: F: 5’-CTCAACTGGTGTCGTGGA-3’, R: 5’-ACTTGTAGCTCAACT-3’; miR-148a-3p: F: 5’-AGCAGTTCAGTGCACTACAG-3’, R: 5’-GCAGGGTCCGAGGTATTC-3’; U6: F: 5’-GCTTCGGCAGCACATATACTAA-3’, R: 5’-AACGCTTCACGAATTTGCGT-3’.

**Dual-luciferase reporter assay**

A Dual Luciferase Reporter Gene Assay Kit (KGAF040, keygen biotech, China) was used to validate the interaction between mRNAs and miRNAs. pGL3 vectors were constructed containing either wild-type (WT) or mutant (Mut) 3’-UTR region with the putative miRNA binding site of mRNA of Sox2 or Runx2. The vectors were then transfected into 293T cells either with or without a miR-200b-3p or miR-130b-3p mimic. The activities of firefly luciferase values were measured 48 h after transfection by a microplate reader (SpectraMax iD5, MD, USA), and were normalized to Renilla luciferase values.

**Treatment of antagomiRs *in vitro***

AntagomiRs and FITC-antagomiRs were purchased commercially (HANBIO, China). For *in vitro* antagomiR treatment, 20 nM ant-NC, ant-200b, or ant-130b was added into the medium and replaced with each medium change.

**Harvest and identification of EVs**

Passage 2-4 BMSCs were cultured in high glucose α-MEM medium with 10 % EV-free FBS (C3801-0100, ViVaCell, China) for 48 h. Then the culture medium was collected and centrifuged at 3000 g for 30 min at 4 ℃ to remove dead cells and debris. Subsequently, the supernatant was centrifuged at 30000 g for 1 h at 4 ℃ to remove big EVs. Next, EVs were isolated from the culture medium by centrifuging at 110000 g for 2 h at 4 ℃ (CP100MX, Hitachi, Japan). The pellets were resuspended in PBS. The EVs were weighed by using the bicinchoninic acid (BCA) method.

**Nanoparticle tracking analysis (NTA)**

The size distribution and concentration of EVs were determined by NTA following the manufacturer's instructions. Briefly, the EVs resuspended and mixed in 30 μL PBS were injected into a particle size analyzer (N30E, NanoFCM, China), and the particle sizes were measured.

**Transmission electron microscopy (TEM) analysis**

The isolated EVs were dropped onto a cupreous electron microscope grid and fixed with 10 μL uranyl acetate at RT for 1min. The samples were observed under a TEM (HT-7700, Hitachi, Japan) at 100 kV.

**Biotin pull-down assay**

Surface proteins of OBPs were labeled with 2 mM EZ-Link Sulfo-NHS-LC-Biotin (A39257, Thermo scientific, USA) at RT for 30 min according to the manufacturer’s instructions. Then proteins of EVs and biotin-labeled OBPs were extracted with a cell lysis buffer for western and immune precipitation (P0013, Beyotime, China). To perform the binding assay, 250 μL biotinylated surface proteins and 250 μL EV proteins were incubated for 4 h at 4 ℃. Next, the mixed complex was incubated with streptavidin magpoly beads (SM01710, Smart-Lifesciences, China) for 30 min at RT. The beads were then washed three times and incubated with elution buffer for 5 min, followed by centrifugation. Eluted proteins were subjected to SDS–PAGE and visualized by coomassie blue staining (P0017F, Beyotime, China).

**Protein identification**

Shotgun proteomics was performed as described [4].

**Lentiviral transfection**

Lentiviral vectors encoding Fn1 were purchased commercially (HANBIO, China). The BMSCs under good culture conditions were seeded into 6-well plates and cultured at 37 ℃ in 5 % CO2 overnight. The BMSCs reaching 30-50 % confluence were used to perform viral infection. Lentiviruses were added into the fresh FBS-free medium at a multiplicity of infection (MOI) of 100 along with 10 μg/ml polybrene. After 24 h, the medium was changed to the fresh FBS-contained medium. Three days after infection, the expression of GFP was observed. Then, the antibiotic selection was performed by adding 0.5 μg/ml puromycin into the medium at each medium replacement for 7 days.

**EVs labeling with DiL and EVs uptake assay *in vitro***

Purified EVs were labeled with the cell plasma membrane staining kit with DiL (C1991S, Beyotime, China). OBPs were grown to 50 % confluence in 12-well plates, and then the medium was replaced with high glucose DMEM containing 5 ng/mL DiL-labeled EVs. After incubation for 48 h, the OBPs were fixed, and nuclei were stained with DAPI.

**Synthesis of EVs-antagomiRs**

To synthesize EVs-antagomiRs, 100 μL 10 mM lipo3000 and 100 nmol ant-NC, ant-200b or ant-130b were mixed and placed for 30 min at RT. Then 50ng EVs diluted in 100 μL PBS was added into the above complexes and shaken at 4℃ overnight. Subsequently, synthetic EVs-antagomiRs were harvested by using ultracentrifugation as described above.

**Treatment of EVs *in vitro***

For *in vitro* EV treatment, 5 ng/mL EVs-NC, EVs-200b, or EVs-130b were added into the medium and replaced with each medium change.

***In vivo* analysis of antagomiR and EVs-antagomiR uptake**

For *in vivo* analysis of antagomiR and EVs-antagomiR uptake, 50 nmol FITC-ant-200b, 50 nmol FITC-ant-130b, 50 ng FITC-labeled EVs-200b, or 50 ng FITC-labeled EVs-130b resuspended in 100 μL PBS was paraperiosteally injected around the right hip of 12-week-old mice weekly for 4 weeks. Then the femurs were collected and sectioned. IF staining for Runx2 was performed to visualize OBPs.

**Western blotting analysis**

Cells or EVs were sonicated into the lysis buffer supplemented with phosphatase and protease inhibitors (KGP2100, Keygen Biotech, China). Proteins were transferred onto PVDF membranes. Then the PVDF membranes were blocked by 5% milk (P0216-1500g, Beyotime, China) and incubated with primary antibodies against GAPDH (1:100000, HRP-60004, proteintech, USA), Sox2 (1:500, A0561, ABclonal, China), P16 (1:1000, ab51243, abcam, UK), Runx2 (1:1000, A2851, ABclonal, China), collagen type I alpha 1 chain (Col1a1)(1:2000, A1352, ABclonal, China), osteocalcin (Ocn)(1:2000, A18241, ABclonal, China), osteopontin (Opn)(1:4000, 22952-1-AP, Proteintech, USA), Fn1 (1:2000, A12977, ABclonal, China), Cd9 (1:1000, ab263019, abcam, UK), Cd81 (1:1000, ab109201, abcam, UK), Tsg101 (1:1000, ab125011, abcam, UK), and Calnexin (1:1000, ab133615, abcam, UK) at 4 ℃ overnight. Membranes were then incubated with goat anti-rabbit IgG(H+L) HRP (70-GAR0072, MultiSciences, China) at 37 ℃ for 1h. Subsequently, the immune complexes were visualized using an Omni-ECL™Enhanced Pico Light Chemiluminescence Kit (SQ101, Epizyme Biomedical Technology, China) and automatic digital gel/chemiluminescence image analysis system (4600SF, Tanon, China).

**Preparation of SA and PF-127 hydrogel**

1 %, 2 %, or 3 % SA solution was prepared by mixing 50 ml double distilled water with 0.5 g, 1 g, or 1.5 g SA, respectively. And 3 % calcium gluconate (CG) solution was prepared by mixing 50 ml double distilled water with 1.5 g CG. SA hydrogel was prepared by slowly dropping CG solution into SA solution immediately before use. 20 %, 25 %, or 30 % PF-127 solution was prepared by mixing 50 ml double distilled water with 10 g, 12.5 g, or 15 g PF-127, respectively. PF-127 hydrogel was prepared by placing PF-127 solution in 37 ℃ immediately before use. Gel time was recorded and TEM was used to observe the pore size of the hydrogel.

***In vitro*** **release of EVs from hydrogel**

To detect the *in vitro* release of EVs from SA and PF-127 with different concentrations, 100 μL 1 % SA, 2 % SA, 3 % SA, 20 % PF-127, 25 % PF-127, or 30 % PF-127 solution mixed with 5 ng DiD-labeled (C1995S, Beyotime, China) EVs was added into 96-well plates. After the solution formed a gel, 100 μL 37 ℃ PBS solution was gently added into the wells. Then the 96-well plates were placed at 37 ℃. At the indicated time points, the supernatant PBS was transferred into a new 96-well plate and imaged using IVIS Lumina Imaging System (IVIS Lumina X5, PerkinElmer, USA).

To detect the *in vitro* release of EVs from hierarchical hydrogel, 3 % SA solution mixed with 5 ng DiL-labeled EVs was added into 96-well plates, and 30 % PF-127 solution mixed with 5 ng DiO-labeled (C1993S, Beyotime, China) EVs was added on the top of the 3 % SA. After the solution formed a gel, 100 μL 37 ℃ PBS solution was gently added into the wells. Then the 96-well plates were placed at 37 ℃. At the indicated time points, the supernatant PBS was transferred into a new 96-well plate and the concentration of DiL-labeled EVs or DiO-labeled EVs in the PBS solution was detected with a microplate reader.

***In vivo* degradation study of hydrogel**

100 μL 3 % SA hydrogel and 100 μL 30 % PF-127 hydrogel was sequentially injected into the backs of nude mice subcutaneously. The remained hydrogel was weighed every 3 days.

**Bioluminescence imaging analysis**

The right hips of nude mice received paraperiosteal injection of 100 μL 3 % SA mixed with 200 ng DiD-labeled EVs and 100 μL 30 % PF-127, while the left hips of nude mice received paraperiosteal injection of 100 μL 3 % SA and 100 μL 30 % PF-127 mixed with 200 ng DiD-labeled EVs in the left GA. Nude mice were imaged using IVIS Lumina Imaging System at the indicated time points.

**Design of animal experiments**

35 12-week-old female mice were randomly divided into sham group (n = 5) and ovariectomy (OVX) group (n = 30). 3 months after surgery, OVX mice were randomly divided into control group, Seq EVs group, Gel group, Gel+EVs-200b group, Gel+EVs-130b group, Gel+EVs-200b/EVs-130b group (n = 5 for each group). For the Seq EVs group, 100 ng EVs-200b resuspended in 100 μL PBS was paraperiosteally injected around the right hip of OVX mice weekly for 2 weeks. After that, 100 ng EVs-130b resuspended in 100 μL PBS was paraperiosteally injected around the right hip of OVX mice weekly for another 2 weeks. For the Gel group, 100 μL SA was paraperiosteally injected around the right hip of OVX mice and 100 μL PF-127 was injected on the outside of the SA immediately. For the Gel+EVs-200b group, 100 μL SA mixed with 200 ng EVs-200b was paraperiosteally injected around the right hip of OVX mice and 100 μL PF-127 mixed with 200 ng EVs-200b was injected on the outside of the SA immediately. For the Gel+EVs-130b group, 100 μL SA mixed with 200 ng EVs-130b was paraperiosteally injected around the right hip of OVX mice and 100 μL PF-127 mixed with 200 ng EVs-130b was injected on the outside of the SA immediately. For the Gel+EVs-200b/EVs-130b group, 100 μL SA mixed with 200 ng EVs-130b was paraperiosteally injected around the right hip of OVX mice and 100 μL PF-127 mixed with 200 ng EVs-200b was injected on the outside of the SA immediately. 4 weeks after injection, the mice in all groups were humanely sacrificed by inhalation of sevoflurane in excess. No chest fluctuation and no heartbeat were the criteria for determining the death of mice. The femurs were collected for subsequent radiological and histological analysis.

**Modeling of OVX-induced osteoporosis**

OVX-induced osteoporotic mice were established as described previously [5]. 12-week-old female mice were normally anesthetized with 100 mg/kg ketamine and 10 mg/kg xylazine by intraperitoneal injection. The bilateral ovaries were exposed via a midline incision in the dorsal skin and two mini-incision in the dorsal skin muscle layer. Then the uterine horns were ligated and the bilateral ovaries were removed. The muscle incision and skin were sutured. As for the mice in the sham group, they were subjected to exposure of bilateral ovaries and incision sutures as described above without the removement of bilateral ovaries.

**Micro computed tomography (μCT) assay**

Samples of femurs were dissected and scanned with high-resolution μCT (Inveon μPET-CT, Siemens, Germany) at a voltage of 80 kVp, 500 μA current, and 15.0 μm resolutions per pixel. An invention research workplace (version 4.2, Siemens, Germany) was used to perform 3D reconstruction analysis. Various parameters of the femoral neck have been applied which included BMD, bone volume/total volume (BV/TV), trabecular number (Tb.N), trabecular thickness (Tb.Th), and trabecular separation (Tb.Sp).

**Preparation of bone tissue sections**

To prepare femur sections, the femur tissues were first fixed with 4 % paraformaldehyde for 24 h and decalcified by using ethylene diamine tetraacetic acid (EDTA) decalcified solution for 4 weeks (G1105, Servicebio, China). Then the femur tissues were embedded into the paraffin and sliced into 5-μm sections.

**Histology assessment**

The femur sections were stained with hematoxylin and eosin (H&E) staining solution (G1005, Servicebio, China) according to the manufacturer’s instructions.

**Immunofluorescence (IF) analysis**

The bone tissue sections and fixed OBPs were blocked with quickblock blocking buffer (P0260, Beyotime, China) for 15 min at RT for subsequent immune staining, followed by incubation with primary antibodies against Ki67 (1:200, A11390, ABclonal, China), proliferating cell nuclear antigen (Pcna)(1:200, A12427, ABclonal, China), Runx2 (1:200, A2851, ABclonal, China), Sox2 (1:200, A0561, ABclonal, China), Col1a1 (1:200, A1352, ABclonal, China), Ocn (1:200, A18241, ABclonal, China), Opn (1:200, 22952-1-AP, Proteintech, USA) at 4 ℃ overnight. Then the sections were labeled with Alexa Fluor594-preabsorbed goat anti-rabbit IgG (ab150084, Abcam, 1:500, UK) or Alexa Fluor 488 AffiniPure F(ab')₂ Fragment Goat Anti-Rabbit IgG (111-546-003, Jackson ImmunoResearch, 1:500, USA) for 2 h at room temperature. Next, the nucleus was counterstained with DAPI. In section-related IF staining, the overlapping fluorescence (Yellow fluorescence) of Runx2 and other antibodies is used to indicate the expression levels of corresponding proteins in OBPs of bone tissues.

**Statistical analysis**

All experiments were repeated three times, and the data were analyzed using GraphPad Prism (9.0, Graph Software, USA). One-way analysis of variance (ANOVA) with Tukey’s post-hoc test for multiple comparisons were applied to determine statistical significance between three groups, while student’s t-tests were applied to determine statistical significance between two groups. To compare the data at different time points, two-way ANOVA and Sidak tests for multiple comparisons was performed. We considered the value of P<0.05 significant. The data were expressed as mean values and standard deviation (SD).

References

1. Liu B, Lu Y, Wang Y, Ge L, Zhai N, Han J. A protocol for isolation and identification and comparative characterization of primary osteoblasts from mouse and rat calvaria. Cell Tissue Bank. 2019;20:173–82. doi:10.1007/s10561-019-09751-0.

2. Ritchie ME, Phipson B, Di Wu, Hu Y, Law CW, Shi W, Smyth GK. limma powers differential expression analyses for RNA-sequencing and microarray studies. Nucleic Acids Res. 2015;43:e47. doi:10.1093/nar/gkv007.

3. Cheng Q, Wang L. LncRNA XIST serves as a ceRNA to regulate the expression of ASF1A, BRWD1M, and PFKFB2 in kidney transplant acute kidney injury via sponging hsa-miR-212-3p and hsa-miR-122-5p. Cell Cycle. 2020;19:290–9. doi:10.1080/15384101.2019.1707454.

4. Tyanova S, Temu T, Cox J. The MaxQuant computational platform for mass spectrometry-based shotgun proteomics. Nat Protoc. 2016;11:2301–19. doi:10.1038/nprot.2016.136.

5. Shen G, Ren H, Shang Q, Zhao W, Zhang Z, Yu X, et al. Foxf1 knockdown promotes BMSC osteogenesis in part by activating the Wnt/β-catenin signalling pathway and prevents ovariectomy-induced bone loss. EBioMedicine. 2020;52:102626. doi:10.1016/j.ebiom.2020.102626.
